# Non-destructive detection of *Phyllostachys edulis* under salt stress using UHF RFID based on Cole-Cole model optimization algorithm

**DOI:** 10.3389/fpls.2025.1678760

**Published:** 2025-10-21

**Authors:** Wen Zhang, Ziyang Hou, Yanyi Liu, Yin Wu

**Affiliations:** The College of Information Science and Technology & Artificial Intelligence, Nanjing Forestry University, Nanjing, China

**Keywords:** *Phyllostachys edulis*, salt stress diagnosis, dielectric properties, C-T-PSO-Cole-Cole hybrid model, UHF RFID

## Abstract

**Introduction:**

Salt stress disrupts cellular osmotic balance in *Phyllostachys edulis*, alters leaf ion distribution and thereby affects dielectric properties. To meet the demand for non-destructive salt stress detection, this study proposes a diagnostic method integrating multi-physics field coupling characteristics.

**Methods:**

Based on the mechanism of salt stress regulating ion concentration in cell sap, a Cole-Cole dielectric model detection framework was constructed by analyzing intrinsic correlations between RFID backscattering signal features and medium dielectric properties. An improved Particle Swarm Optimization (C-T-PSO) algorithm employing Chebyshev chaotic mapping for population initialization and t-distribution dynamic perturbation mechanism was developed to synergistically optimize Cole-Cole model parameters.

**Results:**

Experimental verification showed the C-T-PSO-Cole-Cole hybrid model exceeded 93% in all core metrics (accuracy, precision, recall, F1-score). Comparative experiments with six swarm intelligence optimization algorithms confirmed the model's comprehensive superiority. Convergence curve analysis based on standard test functions demonstrated faster and more stable convergence of the C-T-PSO algorithm. The final model achieved non-destructive diagnosis of salt stress in *P. edulis* using UHF RFID technology with 95.3% accuracy.

**Discussion:**

The hybrid model provides an effective real-time monitoring tool for salinized soil management in bamboo forests, validating the feasibility of salt stress detection through dielectric property analysis.

## 1 Introduction

*Phyllostachys edulis*, as one of the most widely distributed and economically valuable bamboo species in China, plays an indispensable role not only in pulp and paper production, construction, furniture, and landscape architecture, but also demonstrates unique value in maintaining ecological balance and enhancing climate change resilience (Liu et al., 2020; Fu et al., 2024). However, with the increasing severity of soil salinization, salt stress has become a critical environmental factor constraining the growth of *P. edulis* (Zhu et al., 2025). Salt stress disrupts osmotic homeostasis within bamboo cells, significantly alters ion distribution across leaf cell membranes, and consequently affects its dielectric properties and overall physiological function (Singhal et al., 2021). Specifically, salt stress markedly inhibits the growth of *P. edulis* seedlings, manifested as reduced plant height and leaf number, increased leaf curling, and intensified chlorosis. Concurrently, salt stress induces an imbalance in the Na^+^/K^+^ ratio within the plant. Excessive Na^+^ accumulation exerts cytotoxic effects. To combat salt stress, *P. edulis* upregulates the expression of Na^+^/H^+^ antiporter (NHX) genes, compartmentalizing excess Na^+^ into vacuoles to reduce cytosolic Na^+^ concentration (Zhao et al., 2021). Furthermore, salt stress significantly decreases chlorophyll content in seedlings, impairing photosynthetic efficiency and leading to reductions in key photosynthetic parameters such as net photosynthetic rate and stomatal conductance. These alterations not only reveal the detrimental effects of salt stress on *P. edulis* growth but also provide crucial directions for in-depth research into its salt tolerance mechanisms and the development of salt-resistant cultivars.

Traditional methods for monitoring salt stress exhibit significant limitations. Regarding physiological and biochemical indicator measurements, researchers assess the impact of salt stress on *Phyllostachys edulis* by quantifying leaf chlorophyll content, electrolyte leakage rate (Boshimeniuci et al., 2022), proline content, malondialdehyde (MDA) content, and protective enzyme activities. While these metrics directly reflect physiological alterations under salt stress, changes in certain indicators may lag behind the actual stress impact, limiting real-time assessment. In molecular biology techniques, researchers such as Sun Yuanchang and Du Juan have conducted transcriptome sequencing on *Phyllostachys edulis* samples before and after salt stress treatment. This approach identifies differentially expressed genes (DEGs) and analyzes their associated metabolic pathways (Sun et al., 2022; Du et al., 2024). Complemented by metabolomics, this method quantifies stress-induced metabolic fluctuations to reveal remodeling mechanisms in metabolic networks. However, functional validation of identified DEGs remains cumbersome and time-consuming. Recently, electrical signal characterization has emerged as a novel method for plant stress research. Researchers including Zhou Mingu collected electrical signals from salt-stressed *Phyllostachys edulis*, applying signal processing algorithms (e.g., wavelet transform, time-frequency analysis) for noise reduction and feature extraction (Zhou et al., 2019). Analyses of time-domain features (mean, root mean square), frequency-domain characteristics (marginal frequency, centroid frequency), and time-frequency features (wavelet packet energy) reflect physiological impacts of salt stress. While providing a new perspective for stress evaluation, the high cost of precision signal acquisition equipment hinders its widespread adoption.

Traditional methods for salt stress assessment predominantly rely on destructive sampling, hindering dynamic monitoring and limiting data acquisition frequency. Consequently, they fail to comprehensively capture the transient physiological changes and long-term adaptation processes in *Phyllostachys edulis* under salt stress. Furthermore, while controlled laboratory experiments enable precise regulation of salt concentration and stress duration, they struggle to fully replicate the complex and variable environmental conditions encountered in the field. Natural factors such as soil type, microbial communities, and climatic variations cannot be adequately reproduced, thereby compromising the ecological validity of the research findings.

In recent years, non-destructive detection technologies have proliferated, capturing significant research attention. Rubio et al. employed ultra-weak photon emission (UPE) detection technology to investigate the influence of external illumination on fruit UPE by measuring both induced and spontaneous photon emissions from fruits of different colors under natural light and artificial red, green, and blue light. They also conducted a preliminary analysis of photon emission differences between organic and conventional fruits (Rubio et al., 2025). Moghimi et al. achieved quantitative ranking and early detection of salt tolerance in wheat under salt stress by combining hyperspectral imaging with machine learning and image processing methods. This approach not only overcomes the time-consuming and labor-intensive limitations of traditional biomass measurements but also enables rapid assessment of wheat’s response to salt stress as early as 1 day post-treatment, in the absence of visible symptoms (Moghimi et al., 2018). Wu et al. utilized microscopic hyperspectral imaging integrated with chemometric modeling to realize microscopic detection of peroxidase activity in tomato leaves under salt stress. This method avoids the cumbersome steps associated with traditional enzyme activity assays while also providing a visual representation of the spatial distribution of enzyme activity, allowing for real-time monitoring of the physiological responses of leaf cells to salt stress (Wu et al., 2022).

Concurrently, sensor-based diagnostics for plant salt stress are maturing rapidly. Zhang et al. constructed an electrochemical sensor based on MWCNT-Ti3C2Tx-Pd nanocomposites, enabling the detection of hydrogen peroxide release in Arabidopsis leaves under salt stress, thereby providing a novel method for assessing plant stress status (Zhang et al., 2022). The Steinhorst team elucidated a mechanism where excess sodium ions trigger primary calcium signals within specific cell populations in the root tip differentiation zone, forming a “sodium-sensing microenvironment”. This calcium signal transduction pathway subsequently regulates plant salt tolerance (Steinhorst et al., 2022). Furthermore, researchers like Wang et al. developed an *in-situ* soil salinity monitoring system utilizing Wifi POGO electromagnetic sensing technology. By integrating data on soil electrical conductivity and moisture content, this system enables rapid assessment of soil salinization levels in saline-alkali lands, providing crucial environmental monitoring support for plant salt stress diagnosis (Wang et al., 2020).

The evolution of non-destructive detection technologies has catalyzed breakthrough advancements in plant stress diagnostics. Although existing detection methods still face technical bottlenecks such as high cost, limited applicability, insufficient precision, and elevated energy consumption, the rise of Internet of Things (IoT) technology offers new pathways to overcome these challenges. Notably, Radio Frequency Identification (RFID) technology, leveraging its mature IoT application framework, demonstrates significant advantages in agricultural and forestry sectors (Daskalakis et al., 2019). This technology enables non-invasive, real-time status perception and data acquisition from monitored objects through wireless communication between radio frequency signals and electronic tags. Its efficient and precise data processing capabilities provide an innovative solution for non-destructive crop monitoring (Costa et al., 2021).

Against this technological backdrop, several research teams have pioneered innovative applications of RFID technology for salinity measurement. Zhang’s team developed the Plant-Keeper monitoring system, which integrates low-power wireless communication with commercial RFID tags to track plant physiological indicators and biological activities in real-time. This includes monitoring water content and analyzing responses to stressors such as mechanical damage, salinity, low temperature, and drought (Zhang et al., 2021). Hillier’s research group modified commercial RFID sensing tags using polydimethylsiloxane (PDMS) film technology to create reusable passive RFID sensors. These successfully achieved quantitative detection of electrolyte solution concentration changes and demonstrated feasibility for monitoring salt solution concentrations (Hillier et al., 2019). Sharif’s team proposed a low-cost solution based on inkjet printing technology, developing a passive UHF RFID tag detection system. By analyzing variations in tag backscattered power, this system enables rapid detection of salt and sugar content in water bodies (Sharif et al., 2019). In addition to measuring salinity, Wu’s team leveraged UHF RFID technology combined with hyperdimensional computing, collecting leaf tag parameters including RSSI and phase. They constructed a model enabling real-time, high-precision detection of leaf water content (Wu et al., 2024).

Parallel to these advancements, deep learning technologies, particularly Convolutional Neural Networks (CNN) and Long Short-Term Memory (LSTM) networks, have demonstrated remarkable progress in plant stress detection. These approaches excel at automatically extracting complex feature patterns and exhibit outstanding classification accuracy along with powerful end-to-end learning capabilities when applied to large-scale datasets. For instance, Kamarudin et al. developed a lightweight CNN model incorporating an attention mechanism that effectively achieved water stress detection in plants using leaf images (Kamarudin et al., 2023). Azimi et al. employed a CNN-LSTM architecture that successfully captured visual temporal changes induced by water stress throughout the complete growth cycle of chickpeas, enabling early and accurate diagnosis (Azimi et al., 2021). Meanwhile, Wang et al. integrated multi-source sensor data with deep learning networks to accomplish simultaneous high-precision classification of poplar varieties and their drought stress levels (Wang et al., 2024).

Despite the impressive performance of these data-driven methods, their widespread application faces several challenges: Firstly, they typically require large-scale, high-quality datasets for training; Secondly, their decision-making process resembles a “black box” that lacks clear physical interpretability, making it difficult to elucidate the intrinsic physiological mechanisms underlying stress responses; Finally, their complex model architectures present significant challenges for deployment on resource-constrained edge computing devices such as portable field monitors. Specifically regarding RFID signal analysis, while CNNs could effectively process features such as reflected signal strength and phase from tags, and LSTMs would be suitable for modeling the temporal patterns of these parameters, these methods fundamentally rely on learning statistical correlations from data rather than revealing the biophysical principles underlying signal variations.

As a classical dielectric relaxation model, the Cole-Cole model provides a crucial theoretical framework for characterizing the polarization behavior of complex dielectric materials through the frequency-domain response characteristics of complex permittivity. Originally proposed by the Cole brothers in 1941, this model typically manifests as a characteristic semicircular or arc-shaped trajectory in the complex permittivity plane, known as the Cole-Cole semicircle. The geometric characteristics of this arc are intrinsically linked to the polarization mechanisms of the material (Fiandaca et al., 2018; Awal et al., 2024).

Since its inception, the Cole-Cole model has undergone significant development and found extensive application across multiple fields, including biomedical engineering. In biomedical engineering, Lin’s team proposed a method based on a modified Cole-Cole model for detecting trimethylamine N-oxide (TMAO) in early-stage cardiovascular disease. By correlating analysis and computational processing of experimental data, this approach provides a novel pathway for early detection of the disease (Lin et al., 2024). In electromagnetic wave absorption research, Li Jiajun’s team utilized Cole-Cole diagram analysis to characterize the dielectric properties of materials, thereby optimizing the impedance matching design for electromagnetic wave absorbing materials (Li et al., 2024).

Despite the notable successes of the Cole-Cole model across multiple fields, its potential in certain emerging domains remains underexplored. With ongoing refinements to the theoretical model and deepening interdisciplinary research, the Cole-Cole model is poised to play a pivotal role in addressing the complexities of more diverse systems.

**Figure 1:**
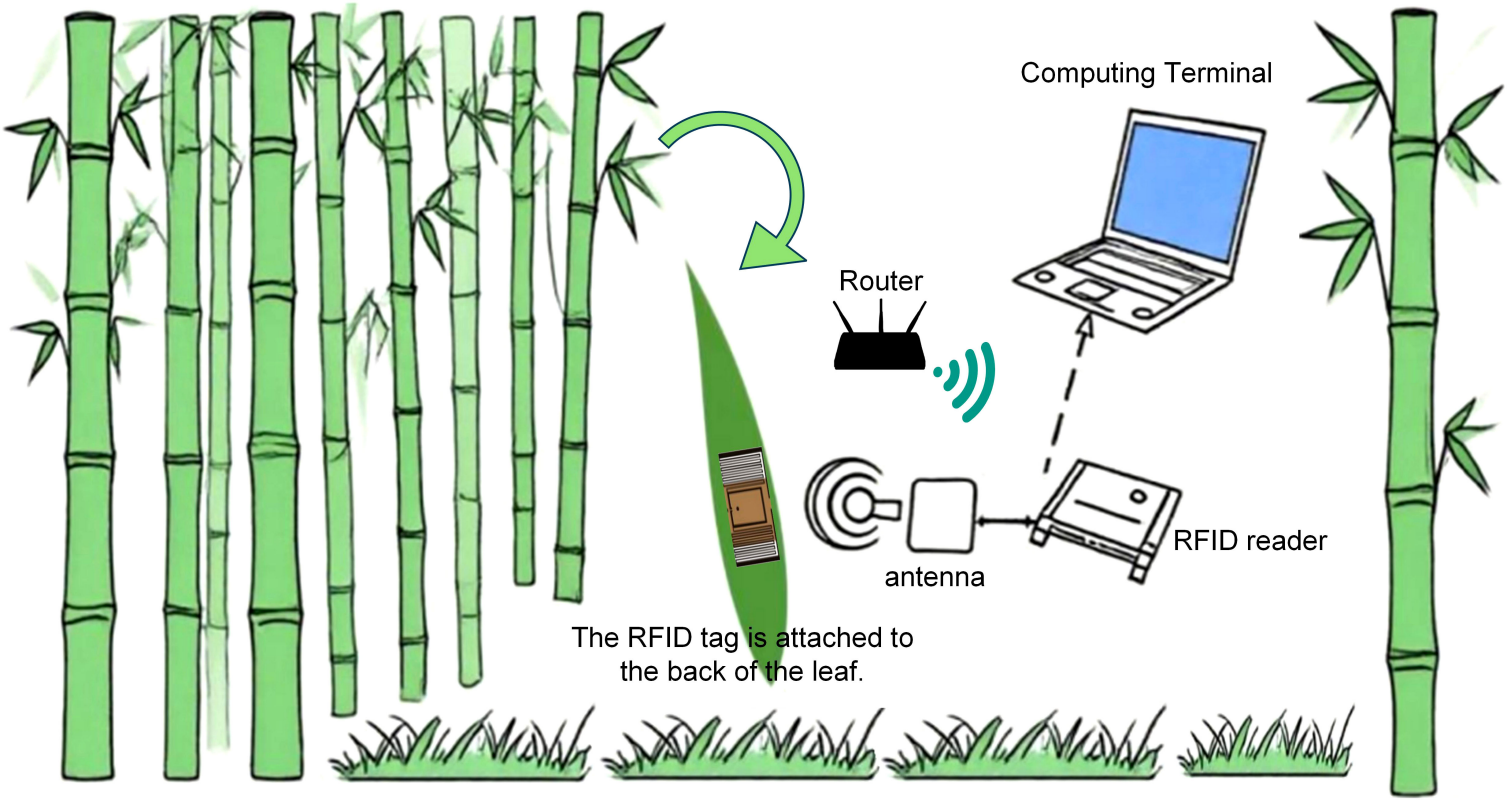
Real-time *in-situ* monitoring system for *Phyllostachys edulis* forests.

To address the challenges of high cost, poor interference resistance, and low stability in salt stress detection for *Phyllostachys edulis*, this study developed a real-time *in-situ* monitoring system for *Phyllostachys edulis* forests based on UHF-RFID technology (**Figure 1**). This system enables rapid and precise acquisition of physiological parameters in complex field environments.

Based on the research objectives, we propose the following preliminary hypotheses and corresponding validation strategies:(1) Salt stress alters the osmotic pressure gradient across leaf blade cell membranes of *Phyllostachys edulis*, thereby modifying its dielectric properties. This dielectric variation can serve as an indicator for quantifying salt stress intensity;(2) UHF radiofrequency signals exhibit high sensitivity to dielectric property fluctuations. By quantifying RF signal characteristics such as RSSI and phase, dynamic variations in the dielectric properties of *Phyllostachys edulis* leaves can be inferred, enabling accurate classification of salt stress levels.

Research Methods: (1) Conduct an in-depth analysis of the mechanism by which salt stress alters osmotic pressure inside and outside cells in *Phyllostachys edulis* leaves, and how this change further affects the dielectric constant of the leaves. Simultaneously, investigate how variations in dielectric properties specifically influence RFID backscatter signals (e.g., phase, RSSI). (2) Dynamically collect backscatter signal data (including key parameters such as RSSI, phase, and read distance) from UHF RFID sensors attached to *Phyllostachys edulis* leaves under different salt stress treatment groups and control groups in laboratory settings. Collect relevant leaf data at the experimental forest base of Nanjing Forestry University. Following data acquisition, precisely calibrate salt stress levels using relative electrical conductivity measurements to validate the accuracy of the algorithmic model.

**Figure 2:**
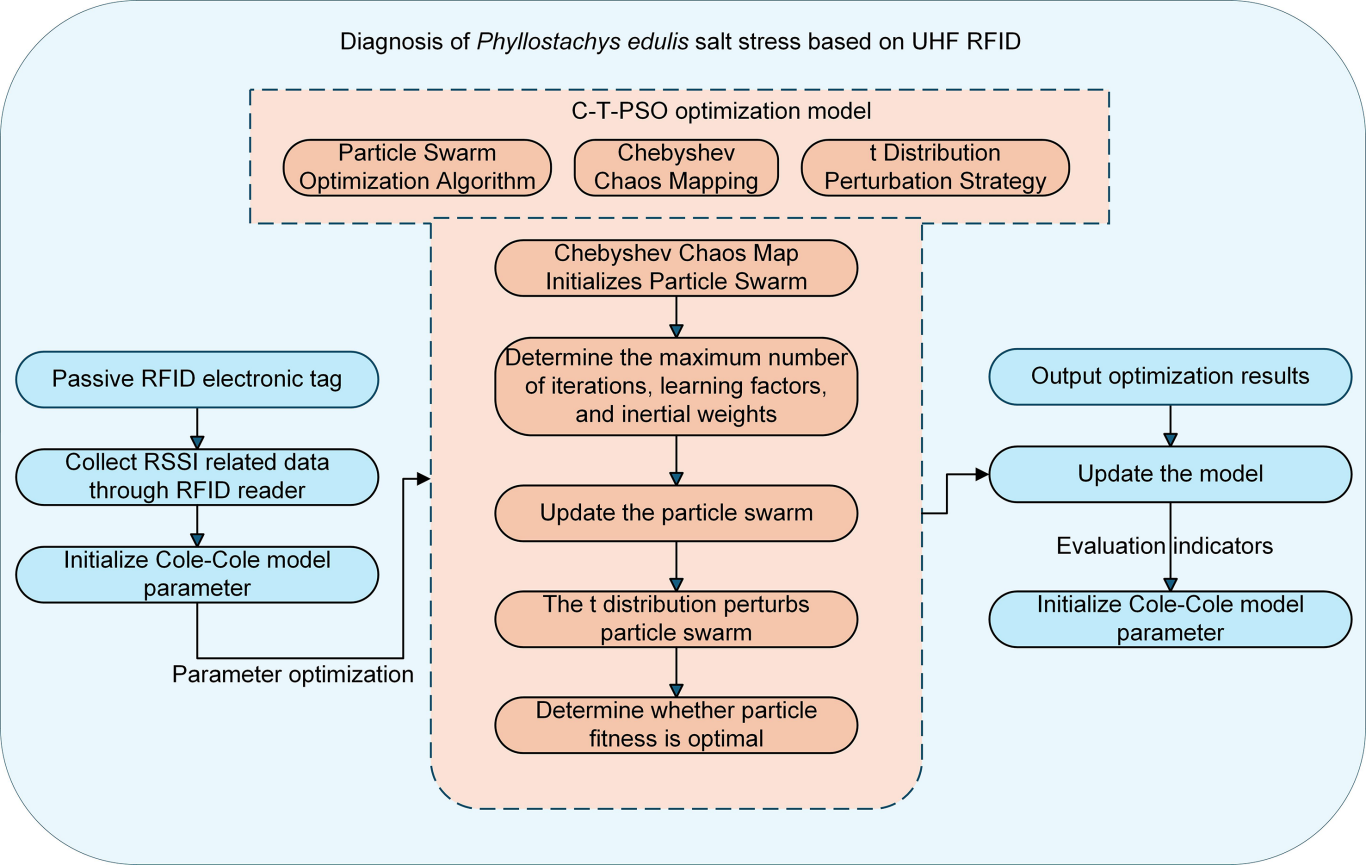
Technical workflow of the UHF RFID-based diagnostic method for salt stress in *Phyllostachys edulis*.

Model Construction: (1) Establish a nonlinear mapping relationship between the dielectric properties of *Phyllostachys edulis* leaves and salt stress levels based on the Cole-Cole dielectric model, elucidating physiological interpretations of model parameters; (2) Incorporate Chebyshev chaotic mapping to initialize the particle swarm (PSO), enhancing global search capability and preventing local optima entrapment; (3) Optimize the PSO algorithm using a t-distribution-based perturbation strategy to dynamically adjust inertia weights, thereby balancing exploration and exploitation capabilities for improved parameter optimization accuracy. The technical workflow of this study is illustrated in **Figure 2**.

## 2 Materials and methods

### 2.1 Relevant theoretical foundations and core principles

#### 2.1.1 UHFRFID communication technology

**Figure 3:**
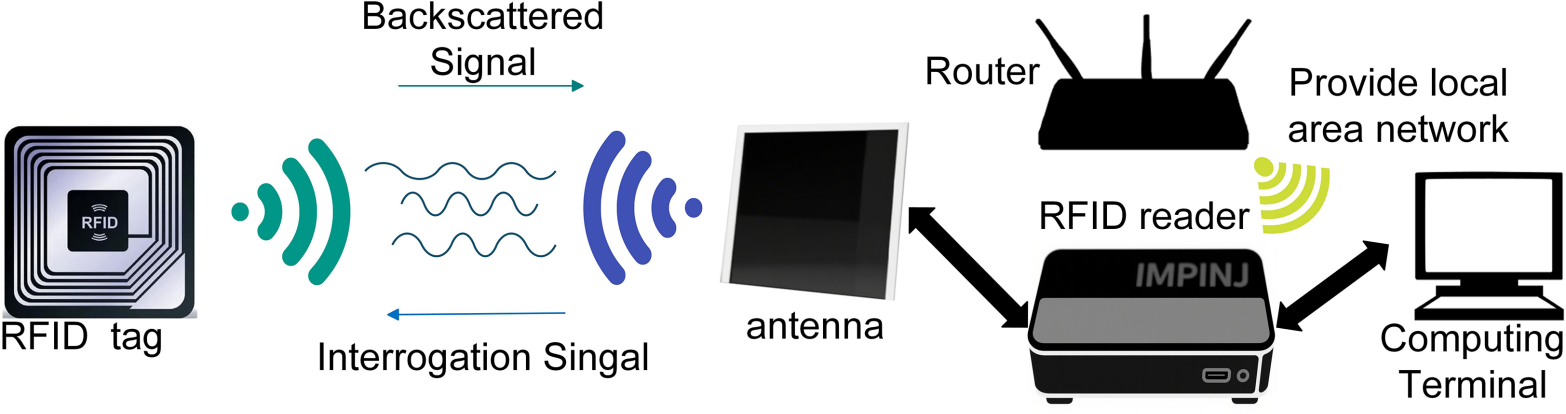
Operating principle of UHF RFID communication.

UHF RFID systems, operating in the 860–960 MHz frequency band, achieve wireless communication primarily through electromagnetic backscatter modulation. Within this system, the reader acts as the active party, continuously radiating a radio frequency electromagnetic field at a specific frequency. Passive tags lack their own power supply and rely entirely on energy coupled from the reader’s emitted electromagnetic field. The key mechanism for information transmission from tags to readers is electromagnetic backscatter modulation (Lee and Jin, 2011). Specifically, the integrated circuit within the tag precisely controls the load state at its antenna port, dynamically altering its reflection cross-section or absorption characteristics relative to incident electromagnetic waves. This load switching primarily employs Amplitude Shift Keying (ASK) modulation, causing systematic variations in the amplitude (corresponding to RSSI) and phase of the signal reflected back to the reader (Xie et al., 2024), thereby encoding data onto the reflected wave. By continuously monitoring Received Signal Strength Indication (RSSI) and phase information, the reader demodulates data transmitted by the tag, with the transmission principle illustrated in **Figure 3**. RSSI directly reflects the strength of received power $(P_{r}$ ), while phase changes exhibit high sensitivity to distance (d), making both critical physical layer parameters for tag distance measurement and localization. Crucially, the physical properties of tagged objects (material, shape, dielectric constant) and variations in the electromagnetic characteristics of the surrounding environment significantly alter the load environment of the tag antenna and its electromagnetic scattering properties, thereby compromising the stability of RSSI and the accuracy of phase measurements.

To quantitatively analyze the fundamental characteristics of the communication link, theoretical modeling is typically conducted under idealized conditions. The assumptions include: free-space propagation, perfect polarization matching, absence of dielectric losses, and ideal port impedance matching at all interfaces. Under these simplified conditions, the signal power at the ultra-high frequency RFID reader’s receiving antenna, denoted as $P_{r}$ (representing the theoretical characterization of RSSI), can be calculated using the following equation (Xie et al., 2024):


(1) $$\begin{array}{c} P_{r}=P_{t}.{\left(\frac{\lambda }{4\pi d}\right)}^{4}.G_{t}.G_{r} \end{array}$$

The parameters in the formula are defined as follows:

-
$P_{t}\;$ denotes the equivalent incident radio frequency power available for modulation at the tag antenna position;
-
$G_{t}$ represents the gain of the tag antenna, affecting energy capture and reflection efficiency;
-
$G_{r}$ indicates the gain of the reader’s receiving antenna, determining the collection capability of weak reflected signals;
-
λ is the operating wavelength of the radio frequency, given by λ=c/f ;

d  is the line-of-sight distance between the tag and reader antenna, governing the attenuation of $P_{r}$ (RSSI) and phase variation $\text{Δ}\phi \approx \frac{4\pi d}{\lambda }\text{mod }2\text{π}$ (Equation 1) reveals that under idealized conditions:

1. RSSI-Based Ranging Principle: The received power $P_{r}$ (RSSI) at the reader is inversely proportional to the fourth power of the distance  d . While distance d can be inferred by measuring RSSI, its accuracy is susceptible to environmental interference;
2. Phase-Based Ranging Principle: The carrier phase variation Δϕ exhibits a linear relationship with distance ( Δϕ∝d ), theoretically enabling higher-precision distance measurement. However, this method suffers from phase ambiguity (cycle slips).

However, real-world application scenarios are far from ideal. Factors such as multipath propagation effects in the environment, obstacle occlusion, eddy current losses in metal objects, energy absorption by high-loss media, electromagnetic noise interference, and antenna mismatch will seriously degrade the correlation between RSSI and distance d , and introduce phase measurement errors. These factors result in actual ranging accuracy being much lower than the theoretical value, which is the core challenge faced by UHF RFID positioning technology.

In this study, we innovatively utilized the unique properties of Ultra-High Frequency Radio Frequency Identification (UHF RFID) technology to conduct in-depth research on salt stress monitoring of *Phyllostachys edulis* leaves. This technology is highly sensitive to changes in the electrical conductivity of media and possesses non-contact penetration detection capabilities, enabling effective reading of data from passive tags attached to the abaxial surface of *Phyllostachys edulis* leaves. By accurately capturing key parameters such as Received Signal Strength Indicator (RSSI), phase shift, and reading distance, and combining them with a dedicated analysis algorithm developed by us, we successfully constructed a quantitative evaluation model for salt stress in *Phyllostachys edulis* leaves. Experimental results demonstrate that this technology can accurately distinguish physiological responses induced by different salt concentration gradients by analyzing the interaction characteristics between electromagnetic waves and leaf electrolyte solutions. It provides a non-destructive, continuous, and high-precision monitoring method for research on salt tolerance of bamboo plants, significantly improving the spatiotemporal resolution of physiological research on salt stress.

#### 2.1.2 RFID backscatter model

The communication foundation of passive RFID systems relies on the interaction mechanism between electromagnetic waves and medium interfaces. When radio frequency waves of a specific frequency emitted by the reader are incident on the surface of plant leaves, their energy distribution follows the boundary conditions described by Maxwell’s equations, resulting in three physical processes: reflection, transmission, and absorption (Benbaghdad et al., 2016). Among them, the intensity of the reflected wave is dominated by the permittivity tensor $\epsilon_{\text{j}}$ , and the reflection coefficient of vertically polarized waves at a specific incident angle ${\text{θ}}_{\text{i}}$ can be quantified using Equation 2:


(2) $$\begin{array}{c} |{\text{Γ}}_{\text{ij}}\left({\text{θ}}_{\text{i}},\epsilon_{\text{j}}\right)|=\frac{{\text{cosθ}}_{\text{i}}-\sqrt{\epsilon_{\text{j}}-\sin^{2}{\text{θ}}_{\text{i}}}}{{\text{cosθ}}_{\text{i}}+\sqrt{\epsilon_{\text{j}}-\sin^{2}{\text{θ}}_{\text{i}}}} \end{array}$$

Here, the imaginary part $\epsilon^{\prime \prime }$ of the complex permittivity $\epsilon_{\text{j}}=\epsilon "-\text{j}\epsilon \text{""}$ satisfies the relationship $\epsilon \text{""}=\text{σ}/\left(\text{ω}\epsilon_{0}\right)$ with the electrical conductivity σ of the leaf tissue cell sap, directly reflecting changes in intracellular Na^+^/K^+^ ion concentrations. This is the physical essence of the electromagnetic response to salt stress. In a monostatic transceiver-integrated architecture, the received power equation derived from the Friis backscattering link model is given by (Equation 3):


(3) $$\begin{array}{c} {\text{P}}_{\text{rx}}=\frac{P_{t}G_{t}^{2}G_{r}^{2}\lambda^{4}{\text{η}}^{2}\cdot |{\text{Γ}}_{\text{ij}}{|}^{2}}{{\left(4\text{πr}\right)}^{4}{\text{ψ}}^{2}{\text{ρ}}^{2}B}M \end{array}$$

In the formula, the wavelength λ and frequency f satisfy λ=c/f ; the polarization loss factor η is determined by the antenna radiation pattern and tag orientation; the gain penalty factor ψ characterizes the wavefront phase distortion caused by leaf curvature (under the Lambertian scattering model, $\text{ψ}\propto 1/\cos^{2}{\text{θ}}_{\text{i}}$ ); the multipath fading margin B needs to compensate for the Fresnel zone diffraction loss of the signal in the plant canopy (typical value: 3–6 dB); the path occlusion factor ρ takes a unit value under line-of-sight propagation conditions. The modulation factor M is determined by the impedance switching strategy of the tag chip, as defined in Equation 4:


(4) $$\text{M}=\frac{1}{4}|{\text{Γ}}_{\text{A}}-{\text{Γ}}_{\text{B}}{|}^{2}$$

where the reflection coefficient ${\text{Γ}}_{\text{A}}$ is defined by the conjugate matching condition in Equation 5:


(5) $$\begin{array}{c} \;{\text{Γ}}_{\text{A}}=\frac{{\text{Z}}_{\text{chip}-}{\text{Z}}_{\text{ant}}^{*}}{{\text{Z}}_{\text{chip}}+{\text{Z}}_{\text{ant}}} \end{array}$$

When using Phase Shift Keying (PSK) modulation, the chip only changes the load reactance component (ΔX ≠ 0, ΔR = 0), enabling phase jumps of Γ while maintaining stable DC power supply. In contrast, Amplitude Shift Keying (ASK) alters the reflection amplitude through switching of the resistance component (ΔR ≠ 0), which requires a trade-off between modulation depth and the chip’s turn-on voltage. The Received Signal Strength Indicator (RSSI), as a direct observable of the system, follows a logarithmic law in its conversion to received power, as expressed in Equation 6:


(6) $$\begin{array}{c} RSSI=20\log_{10}(\frac{\text{P}}{10^{-3}})dBm\left(P:mW\right) \end{array}$$

The hardware parameters and dielectric response terms can be decoupled by combining the equations, yielding the expressions in Equations 7, 8:


(7) $$\begin{array}{c} {\text{P}}_{\text{rx}}=K\cdot |{\text{Γ}}_{\text{ij}}{|}^{2}\left(K=\frac{P_{t}G_{t}^{2}G_{r}^{2}\lambda^{4}{\text{η}}^{2}M}{{\left(4\text{πr}\right)}^{4}{\text{ψ}}^{2}{\text{ρ}}^{2}B}\right) \end{array}$$

(8) $$\begin{array}{c} RSS{\text{I}}_{\text{rx}}=10\log_{10}\text{K}+20\log_{10}|{\text{Γ}}_{\text{ij}}|+30 \end{array}$$

Salt stress causes changes in the permeability of leaf cell membranes. The influx of extracellular Na^+^ increases the electrical conductivity σ by 20-100%, leading to a significant increase in ϵ"" in the 0.5–5 GHz frequency band. According to the above equation, when ϵ"">5 , $|{\text{Γ}}_{\text{ij}}|$ can be enhanced by 8–15 dB at an incident angle of 60°, ultimately resulting in distinguishable stress response characteristics in RSSI readings. This phenomenon lays the electromagnetic theoretical foundation for passive radio frequency monitoring of plant physiological states. Integrating the above electromagnetic transmission model and modulation mechanism, under the constraints of fixed system antenna parameters ( ${\text{G}}_{\text{t}},{\text{G}}_{\text{r}},\text{η},\text{ψ}$ ) and operating distance, the backscattered power received by the reader can be characterized as a single-valued function of the complex permittivity $\epsilon_{\text{j}}$ of the medium.

#### 2.1.3 Cole-Cole dielectric model

The previous section of the research confirmed that the power attenuation and phase shift of RFID backscattered signals can sensitively reflect changes in the dielectric properties of plant leaves. Plant tissue, as a heterogeneous biological dielectric, undergoes significant changes in its cellular structure, water content, and ion concentration under salt stress, which directly affects the propagation characteristics of electromagnetic waves. To quantify this relationship, it is necessary to establish an accurate dielectric model. The classic Debye model describes a single relaxation polarization process, as shown in Equation 9 (Benbaghdad et al., 2016):


(9) $$\begin{array}{c} \epsilon \left(\omega \right)=\epsilon_{a}+\frac{\epsilon_{0}-\epsilon_{a}}{1+j\omega \tau } \end{array}$$

In the equation, ${\text{ϵ}}_{\text{a}}$ and ${\text{ϵ}}_{0}$ represent the limiting dielectric constants at optical frequency (>100 GHz) and electrostatic field (0 Hz) respectively, τ is the dipole rotation relaxation time, and ω is the angular frequency. This model is applicable to ideal polar liquids, but it cannot characterize the widely distributed relaxation time spectrum of biological tissues.

To address this limitation, the Cole-Cole model introduces a relaxation time distribution parameter α ( α(0≤α<1) ), and its general expression is given by Equation 10:


(10) $$\begin{array}{c} \epsilon \left(\omega \right)=\epsilon_{a}+\sum_{i=1}^{n}\frac{\epsilon_{0}-\epsilon_{a}}{1+{\left(j\omega \tau_{i}\right)}^{1-\alpha_{i}}}+\frac{\sigma_{dc}}{j\omega \epsilon_{v}} \end{array}$$

When ${\text{α}}_{\text{i}}$ =0 in the equation, the model degenerates into the Debye model; an increase in ${\text{α}}_{\text{i}}$ indicates enhanced dispersion of time constants in the relaxation process. This model quantifies multiple mechanisms such as cell membrane interfacial polarization and protein dipole relaxation through ${\text{ α}}_{\text{i}}$ , making it particularly suitable for the water-biomacromolecule composite system in leaves.

The complex permittivity ${\text{ϵ}}^{*}$ and conductivity satisfy the following relationship described in Equation 11:


(11) $$\begin{array}{c} \epsilon^{*}\left(\omega \right)=\epsilon '\left(\omega \right)-j\frac{\sigma \left(\omega \right)}{\omega \epsilon_{0}} \end{array}$$

Salt stress affects this equation through two pathways: the intracellular accumulation of Na^+^/Cl^+^ ions significantly increases the static conductivity ${\text{σ}}_{\text{dc}}$ ; meanwhile, cellular dehydration leads to a reduction in free water, resulting in a decrease in the low-frequency permittivity ${\text{ ϵ}}_{0}$ and an extension of the relaxation time τ. These two types of changes in dielectric response collectively constitute the electromagnetic characteristics of the stress state.

The direct current conductivity ( ${\text{σ}}_{\text{dc}}$ ) of plant leaves exhibits significant responsive characteristics to salt stress, which can serve as a key bioelectrical indicator for characterizing the stress state. Through the coupling analysis of the Cole-Cole dielectric model and RFID backscattering power, the transfer relationship of “electromagnetic scattering signal—leaf complex permittivity—conductivity” has been successfully established. To balance model accuracy and computational complexity, this paper adopts the first-order Cole-Cole model (n=1). This simplified form can still effectively capture the α parameter shift and ${\text{σ}}_{\text{dc}}$ dynamic changes caused by salt stress in the characteristic relaxation frequency band of plant tissues.

#### 2.1.4 Improved C-T-PSO-Cole-Cole multi-strategy optimization algorithm

To balance model accuracy and computational complexity, this paper adopts the first-order Cole-Cole model (n=1), formulated in Equation 12:


(12) $$\begin{array}{c} \epsilon \left(\omega \right)=\epsilon_{\infty }+\frac{\epsilon_{0}-\epsilon_{\infty }}{1+{\left(j\omega \tau_{i}\right)}^{\left(1-\alpha \right)}}+\frac{\sigma_{s}}{j\omega \epsilon_{v}} \end{array}$$

The accurate identification of key parameters (α, τ, ${\text{ ϵ}}_{0}$ , ${\text{ ϵ}}_{\infty }$ ) in the first-order Cole-Cole dielectric model (Eq. 12) is paramount for high-precision dielectric spectrum reconstruction. This study employs an enhanced Particle Swarm Optimization (PSO) algorithm for the joint inversion of these parameters. The physical significance and optimization bounds of each parameter are detailed in **Table 1**.

**Table 1:** Optimization parameters of the Cole-Cole model.

Parameter name	Parameter meaning	Find the best scope
α	The distribution characteristics of the control response affect the behavior of the dielectric constant at different frequencies	(0,1)
τ	Timescale for controlling dielectric response	[0.1,2]
$\epsilon_{0}$	Dielectric response of materials at relatively low frequencies (860MHz)	[2,60]
${\text{ϵ}}_{\infty }$	Dielectric response of materials at relatively high frequency (960MHz)	[1,25]

This study is built upon the standard PSO algorithm framework (Stehlik et al., 2024), which facilitates efficient search in the parameter space by simulating collective swarm intelligence. Particles update their velocity and position based on their individual historical best position ( ${\vec{P}}_{\text{best}}$ ) and the swarm’s global best position ( ${\vec{G}}_{\text{best}}$ ). However, the standard PSO is often prone to premature convergence and limited population diversity when solving such complex inverse problems.

To overcome these limitations, two key enhancements are proposed. Firstly, a Chebyshev chaotic map is adopted for population initialization (Zhang et al., 2025). The initial solutions are generated using (Equation 13) and then mapped to the parameter search space via the affine transformation in (Equation 14):


(13) $$\begin{array}{c} x_{n+1}=\cos\left(n\cdot \arccos\left(x_{n}\right)\right) \end{array}$$

(14) $$\begin{array}{c} \;X_{i}=\frac{\left(x_{n}+1\right)}{2}\left(\;X_{\max}-X_{\min}\right)+X_{\min} \end{array}$$

Compared to traditional random initialization, this method leverages the ergodicity, randomness, and sensitivity to initial conditions inherent in chaotic systems to produce an initial population with superior dispersion. This establishes a better foundation for subsequent global search and effectively suppresses premature convergence.

Secondly, a t-distribution perturbation strateg**y** is incorporated during the later iterations. This mechanism enhances population diversity by injecting a t-distributed random perturbation into the current global best solution ( ${\vec{G}}_{\text{best}}$ ), aiding the algorithm in escaping local optima. Its mathematical formulation is given by (Equation 15):


(15) $${\vec{P}}_{new}^{best}={\vec{G}}_{best}+\delta \cdot \vec{T}(v)$$

where $\vec{T}(v)$ is a D-dimensional t-distributed random vector and δ is a scaling factor. The heavy-tailed nature of the t-distribution (particularly when the degrees of freedom parameter ν is small) enables the generation of larger exploration steps compared to Gaussian perturbations. By adjusting ν, this strategy can adaptively balance the algorithm’s requirement for global exploration and local exploitation during different iteration stages.

The algorithm’s hyperparameters were configured based on theoretical and experimental validation as follows: a population size of N=50 was selected to maintain population diversity while controlling computational complexity; an inertia weight of w=0.7 was set to effectively balance global exploration and local exploitation; learning factors c1=c2=2.0 were adopted following the standard cognitive-social model to ensure equilibrium between individual experience and swarm intelligence; a perturbation scaling factor of δ = 0.5 was determined through testing to optimally balance exploration of new regions and search stability; a maximum iteration count of $M_{max}=1000$ was established based on solution space complexity analysis, satisfying convergence requirements in over 99% of cases while incorporating an early stopping mechanism to prevent unnecessary iterations; random numbers ${\text{r}}_{1}$ and ${\text{ r}}_{2}$ were set to follow a U(0,1) uniform distribution to preserve stochasticity and exploratory behavior throughout the iterative process.

**Figure 4:**
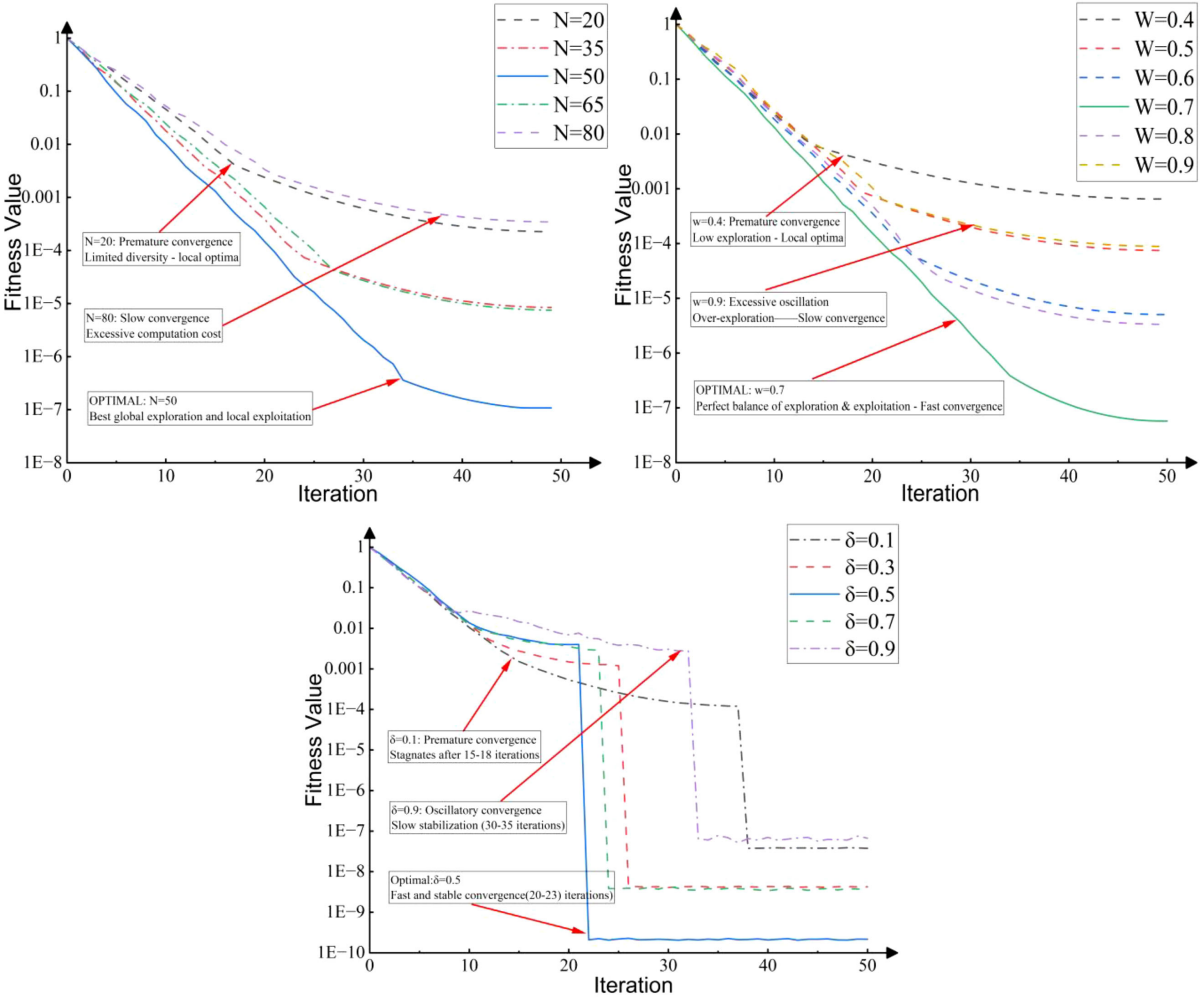
Sensitivity analysis of key parameters.

The hyperparameters of the C-T-PSO algorithm were initially determined based on empirical conventions and preliminary experiments. To further validate the robustness and generalizability of these choices, a comprehensive sensitivity analysis was conducted on three key parameters: population size (N), inertia weight (w), and scaling factor (δ). As shown in **Figure 4**: Sensitivity analysis of key parameters.

The analysis was performed using a controlled variable approach on benchmark functions, monitoring the convergence accuracy and speed. This analysis confirms that the empirically selected parameter set is robust and generalizable, providing stable performance across different optimization landscapes.

#### 2.1.5 Classification model

The specific steps of the classification method are as follows:

(1) Use an RFID reader to collect relevant information of *Phyllostachys edulis* at different salt stress stages, and expand the collected data into a model dataset. (2) Perform conversion processing on the data in the dataset, complete sample labeling, and generate training sample sets required by the model. (3) Randomly sample the generated sample sets and divide them into training sets, test sets, and validation sets in a ratio of 7:2:1. Then, input the training sets into the model for training. (4) Adjust the model parameters based on its performance on the test sets, and then retrain the model. Repeat this process until the performance on the test sets reaches the optimal level, indicating that the model has converged to an ideal state. (5) Use the trained and optimized model as a detector to detect salt stress in *Phyllostachys edulis* and determine the specific type of salt stress.

To address the challenge of limited sample size and to more robustly evaluate the generalization performance of the classification model, a k-fold cross-validation analysis was supplemented in this study. The specific process was as follows: the entire dataset was randomly partitioned into 10 mutually exclusive subsets (folds). Sequentially, one subset was used as the test set, and the remaining nine subsets were combined as the training set. The training and testing process was repeated 10 times. The final performance metrics of the model are presented as the mean and standard deviation of the results from these 10 tests. This analysis aims to validate the stability of the results obtained based on a single split.

### 2.2 experimental preparation

#### 2.2.1 Seedling cultivation and pretreatment

One-year-old healthy *Phyllostachys edulis* seedlings (with a height of 30 ± 2 cm) available in the market were selected, ensuring they had a consistent genetic background and were free from pests and diseases. Polyethylene pots with a diameter of 25 cm and a height of 30 cm were used as cultivation containers. The substrate was mixed in a volume ratio of vermiculite: peat soil: perlite = 3:1:1. This formula has both water retention and air permeability. The initial pH value was adjusted to 5.5-6.0 (using 0.1 mol/L HCl or NaOH), which is in line with the characteristic of *Phyllostachys edulis* preferring slightly acidic soil. Before transplantation, the root systems of the seedlings were soaked in a 0.1% carbendazim solution for disinfection for 10 minutes, then washed clean and planted in the substrate, with one seedling per pot.

The seedlings were acclimatized for 14 days in an artificial climate chamber under the conditions of temperature 25 ± 2°C, relative humidity 70 ± 5%, and light intensity 300μmol·m^+^²·s^+^¹ (photoperiod 12 h/d). They were watered with Hoagland nutrient solution daily to promote root recovery.

#### 2.2.2 Salt stress treatment design

Six treatment groups were set up: control group (CK, 0 mmol/L NaCl), T1 (25 mmol/L NaCl), T2 (50 mmol/L NaCl), T3 (75 mmol/L NaCl), T4 (100 mmol/L NaCl), T5 (125 mmol/L NaCl), and T6 (150 mmol/L NaCl). Each group was replicated in 6 pots, arranged in a completely randomized block design. After acclimatization, the stress treatment was initiated: a 50 mL medical syringe was used to inject NaCl solution into the main root distribution area (5–10 cm in depth), with 20 mL injected per plant, twice a week (at 3-day intervals) for 4 consecutive weeks.

The experiment was conducted in a controlled greenhouse, where the temperature was maintained at 28 ± 2°C during the day and 22 ± 2°C at night, with an air humidity of 70 ± 5%. Natural light was supplemented with LED lighting (light intensity 400-600μmol·m^+^²·s^+^¹). A soil temperature and moisture transmitter (DHT11) was placed in each pot to measure air temperature and humidity, and the relevant data were transmitted to the cloud platform in real time for data management. This ensured that the temperature remained within an appropriate range, and the substrate water content was maintained at 60% ± 5% of the normal water content to avoid interference from drought.

#### 2.2.3 Hardware composition and equipment selection of the data acquisition system

**Figure 5:**
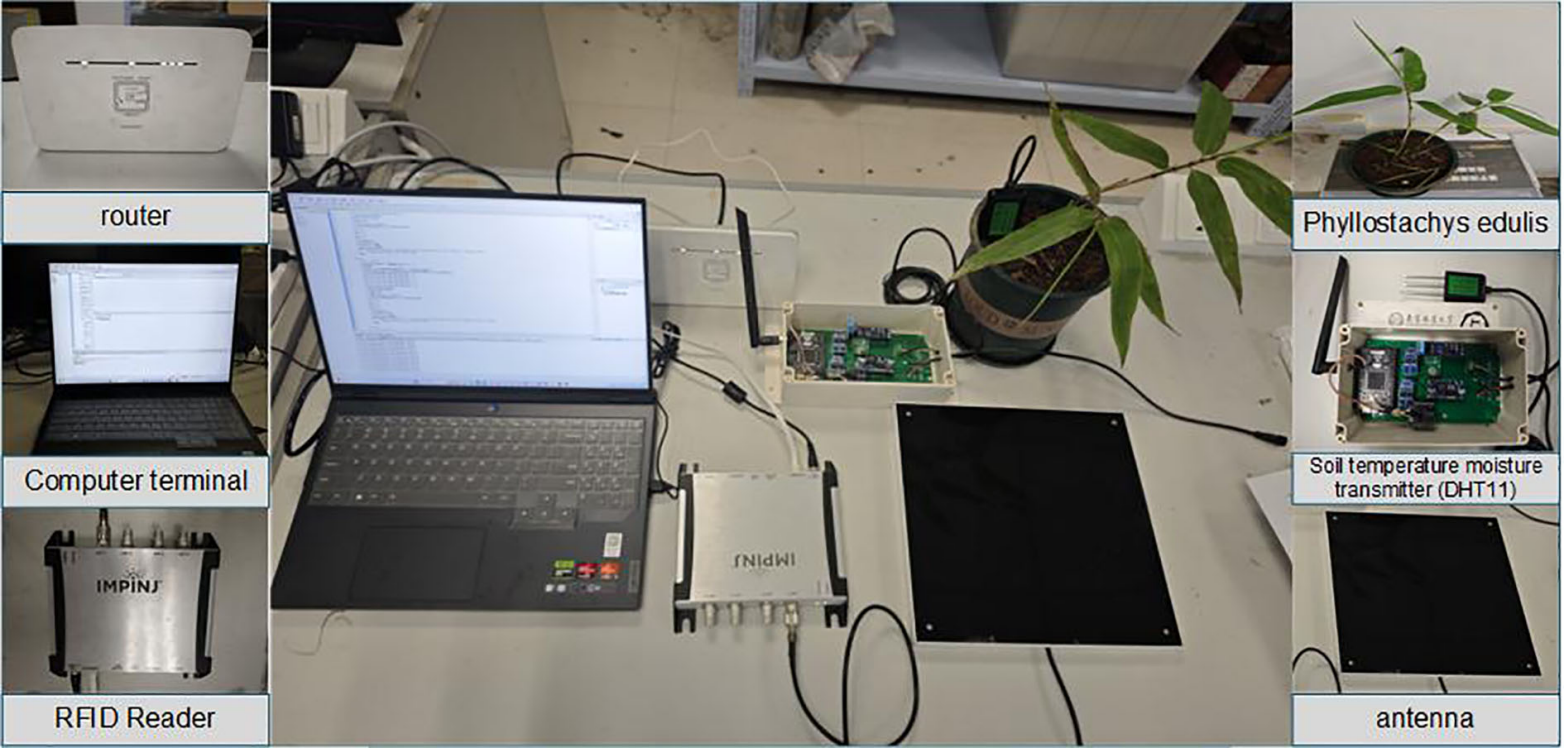
Hardware devices of the data acquisition system.

The hardware of the data acquisition system mainly includes RFID tags, a reader, and an antenna (**Figure 5**). The antenna is connected to the reader via a feeder line. The system is powered by a mobile power supply, and a router provides a local area network to ensure communication between the reader and the computer terminal.

**Figure 6:**
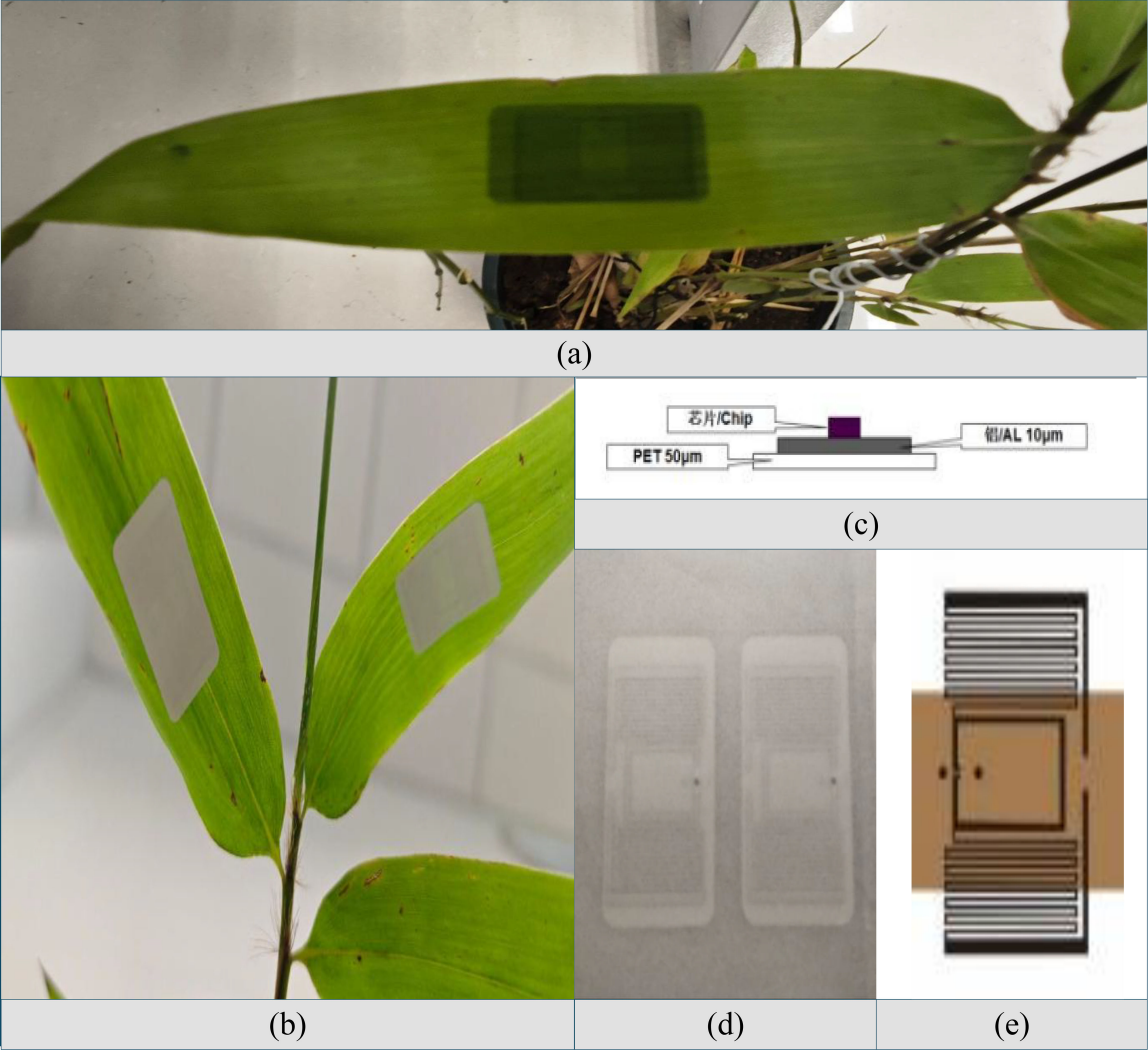
Lingtian KU7 passive electronic tag. **(a)** Electronic tags are pasted on the front view of *Phyllostachys edulis*. **(b)** Electronic label attached to the back of *Phyllostachys edulis*. **(c)** Schematic diagram of the tag's material structure. **(d)** Physical image of the electronic inlay. **(e)** Dimensional schematic of the tag.

(1) Reader: The Impinj Speedway R420 high-performance UHF RFID reader is adopted. This device supports mainstream protocols such as EPC Global Gen 2 and ISO 18000-6C, and provides multiple interfaces including PoE, RS-232, and USB, facilitating system integration;
(2) Antenna: The RYT-280 circularly polarized antenna is selected, which is responsible for signal transmission and reception. With a moderate size and high gain characteristics, this antenna is suitable for working in the ultra-high frequency band;
(3) RFID tag: The Lingtian KU7 passive electronic tag was used, as shown in **Figure 6**. Based on the traditional wet inlay, the tag is encapsulated with PET flexible material and has a symmetrical rectangular structure, which conforms to the basic shape of *Phyllostachys edulis* leaves. Its 3M adhesive ensures firm attachment and prevents edge curling and falling off, and the PET material also provides excellent waterproof performance.

#### 2.2.4 Design and implementation of the software-side data acquisition program

On the software side, the host computer uses the Impinj Octane SDK to process the radio frequency signals collected by the reader and extract parameters such as the tag EPC number, RSSI (signal strength), phase, collection time, antenna port number, and frequency. A data acquisition program was developed based on the Java interface of this SDK, which controls the reader to perform operations by instantiating an Impinj Reader class object. The specific process includes: (1) Connect the host and the reader to the same local area network through a specified IP address to establish a connection; (2) Before starting, configure the reader’s reading mode to MaxThroughput; use the ReportConfig class to set the data items to be collected (in addition to the default EPC, explicitly specify the need to collect RSSI, phase, time, antenna number, and frequency, and control the collection of each signal attribute through flag bits); (3) Configure the antenna to operate in single-antenna mode, set the transmission power to 25.0 dBm and the reception sensitivity to -70.0 dBm to ensure signal stability; (4) Start the reader after completing the configuration. Data collection continues for a fixed 10 seconds, then automatically stops and disconnects; (5) To suppress signal interference (collision) caused by simultaneous responses from multiple tags, set a tag mask filter to restrict the reader to accessing only tags within a specified EPC range. This program achieves efficient and accurate data acquisition and analysis functions, making it particularly suitable for complex environments with multiple tags.

#### 2.2.5 Data collection process

**Figure 7:**
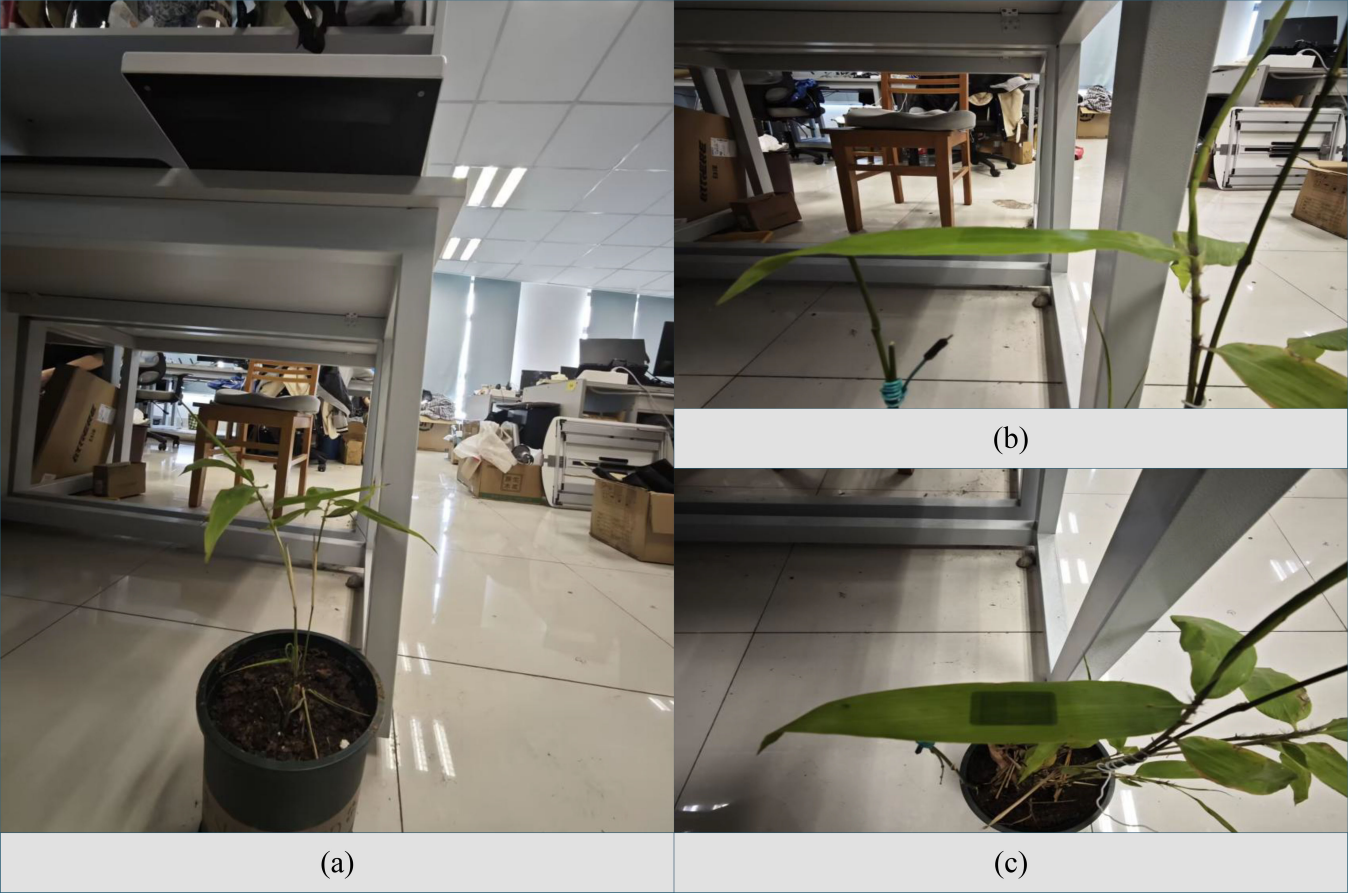
Data acquisition perspectives. **(a)** Frontal overview of the experimental setup, showing the relative positions of the plant, antenna, and reader. **(b)** Close-up view detailing the method of immobilizing the *Phyllostachys edulis* leaves. **(c)** Side view illustrating the fixed angle and distance between the antenna and the target leaf.

To accurately measure the RSSI value scattered by the RFID tag on the back of *Phyllostachys edulis* leaves, it is necessary to ensure that the leaves remain stationary during the measurement and that their relative angular position with respect to the reader antenna remains stable. Therefore, the experimental setup is as shown in **Figure 7**, where both the RFID reader and *Phyllostachys edulis* leaves are placed in a fixed position. This setup, on the one hand, meets the requirements of measurement accuracy; on the other hand, it is designed to ensure that the radio frequency signals reflected by the RFID tag attached to the back of the leaf can effectively penetrate the leaf tissue and be reliably received by the reader antenna.

To balance the accuracy and efficiency of data collection, a phased dynamic sampling strategy was adopted in this experiment: In the initial observation phase, within one hour, the RFID reader collected data every 6 minutes, with 300 sets of data collected each time to comprehensively capture the characteristics of dynamic changes; once the system state tended to be stable, it entered the regular monitoring phase, where the sampling interval was adjusted to 30 minutes per time, with 300 sets of experimental data collected each time. This ensures data timeliness while optimizing the allocation of storage resources.

#### 2.2.6 Determination of relative electrical conductivity of *Phyllostachys edulis* leaves

**Figure 8:**
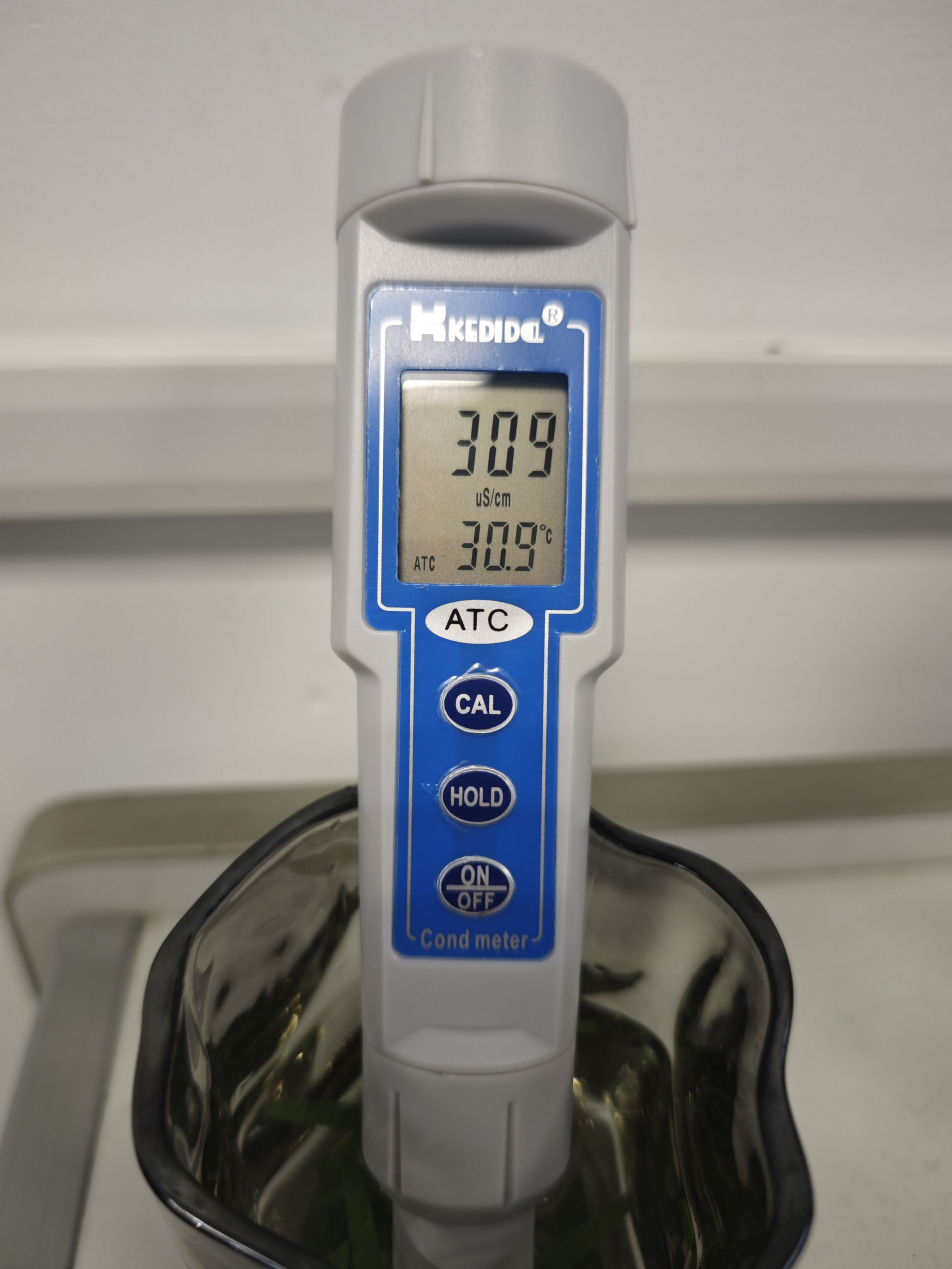
TDS Pen.


To evaluate the degree of environmental stress on *Phyllostachys edulis* leaves, this study used relative conductivity (RC) as an indicator for determination. The principle is based on the increased electrolyte leakage caused by cell membrane damage. Functional leaves of *Phyllostachys edulis* were selected, rinsed with deionized water, and surface moisture was blotted dry. A punch was used to sample leaves while avoiding the main veins, and leaf samples of equal area (approximately 0.1 g) were weighed. The samples were placed in stoppered test tubes, 20 mL of deionized water was added, and they were soaked at room temperature for 1 hour. A conductivity meter (TDS pen) was used to measure the conductivity value C_1_ of the leaf soaking solution at this point. Subsequently, the test tubes were placed in a boiling water bath and boiled for 30 minutes to kill the tissues. After shaking and cooling, the final conductivity value C_2_ was measured. The conductivity meter (TDS pen) is shown in **Figure 8**. The relative conductivity (RC) of the leaves was calculated according to (Equation 16) (Cao et al., 2025):

(16) $$\begin{array}{c} RC=\frac{{\text{C}}_{1}}{{\text{C}}_{2}}\times 100\% \end{array}$$

The salt stress status of *Phyllostachys edulis* leaves is inferred by analyzing the RSSI values of RFID tags. The RFID reader is used to read the tags attached to the back of the leaves, obtain their RSSI signals, and specific algorithms are employed to process these signals to determine the stress level.

#### 2.2.7 Data collection related to salt stress in *Phyllostachys edulis* leaves

**Figure 9:**
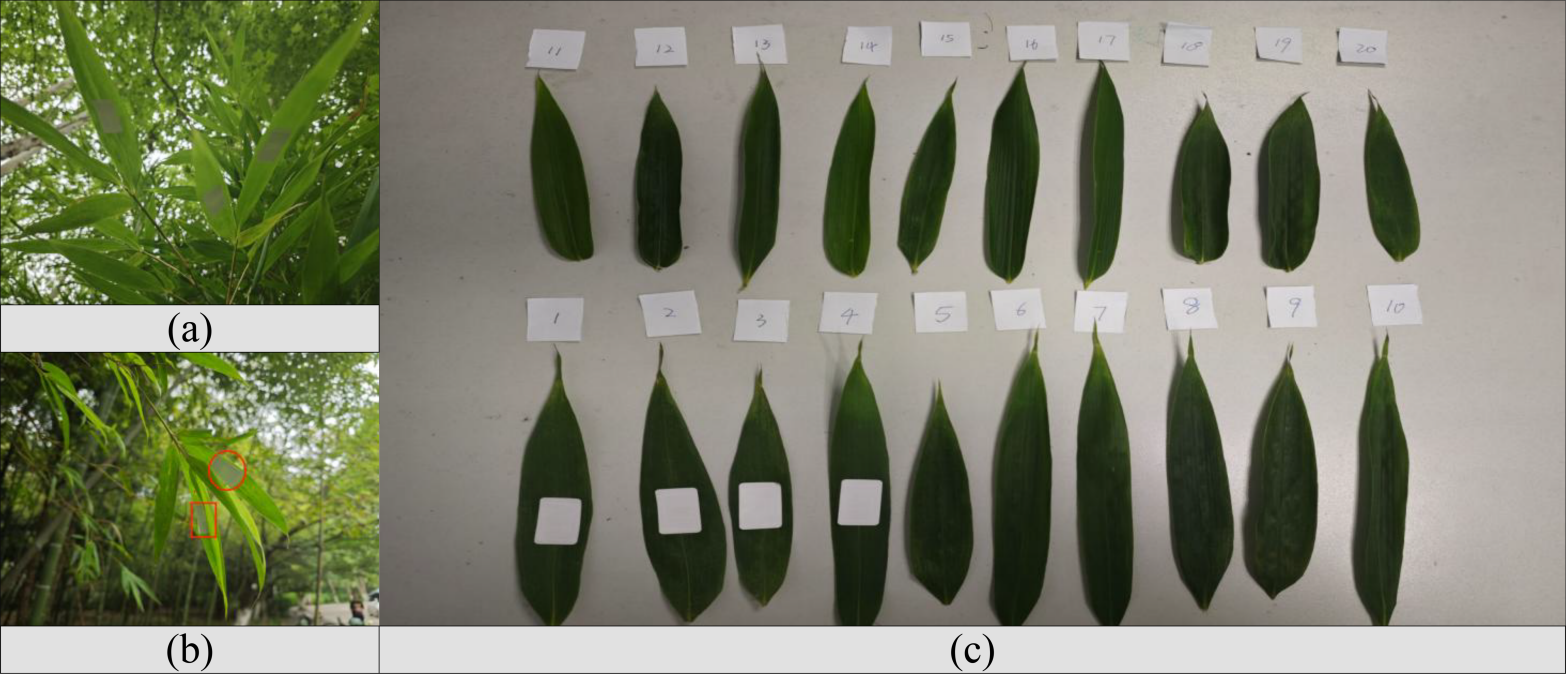
On-site collection and preparation of bamboo leaf samples. **(a)** Bamboo leaves in their natural field environment prior to collection. **(b)** Tagging locations indicated by red markers on the bamboo stems. **(c)** Collected leaf samples arranged and numbered for subsequent analysis.

At the experimental forest base of Nanjing Forestry University, several bamboo leaves were selected. RFID tags were attached to the back of the leaves on-site, and a fixing device was secured (to ensure signal transmission). Data such as RSSI and phase of the tags were collected using an RFID reader and immediately saved, as shown in **Figure 9**. The leaves were then promptly collected, rinsed with deionized water and blotted dry. Samples of approximately 0.1 g were punched out, avoiding the main veins, and placed in stoppered test tubes with 20 mL of deionized water. After soaking at room temperature for 1 hour, the tubes were heated in a boiling water bath for 20 minutes, shaken, and cooled to room temperature. A conductivity meter was used to measure the conductivity of the soaking solution and the cooled boiled solution. The relative conductivity was calculated according to the formula. Finally, the data were input into the algorithmic model to predict the stress status, and the error rate was analyzed by comparing with the measured results to verify the model’s accuracy.

#### 2.2.8 Evaluation indicators

To objectively evaluate the performance of the *Phyllostachys edulis* salt stress detection model constructed in this study, Accuracy, Precision, Recall, and F1-Score were selected as core evaluation metrics (Chetry et al., 2025). These metrics quantify the model’s predictive efficacy for leaf salt stress states (e.g., no stress, mild salt stress, moderate salt stress, severe salt stress) from different dimensions. The definitions and calculation methods of each metric are shown in **Table 2** below:

**Table 2:** Model evaluation index.

Metric Name	Calculation formula	meaning
Accuracy	$\text{Accuracy}=\frac{\text{TP}+\text{TN}}{\text{TP}+\text{TN}+\text{FP}+\text{FN}}$	The correct proportion of the model predicts the sample
Precision	$\text{Precision}=\frac{\text{TP}}{\text{TP}+\text{FP}}$	The proportion of the predicted positive category is actually positive category
Recall	$\text{Recall}=\frac{\text{TP}}{\text{TP}+\text{FN}}$	The proportion of the positive category that is actually predicted to be positive category
F1-Score	$\text{F}1-\text{Score}=2\times \frac{\text{Precision }\times \text{Recall}}{\text{Precision}+\text{ Recall}}$	The harmonic mean of precision and recall, comprehensively balancing both metrics to reflect overall model performance

TP (True Positive) refers to the number of samples that actually belong to a certain level and are correctly predicted; TN (True Negative) refers to the number of samples that do not actually belong to a certain level and are predicted to be of other levels; FP (False Positive) refers to the number of samples that do not actually belong to a certain level but are mistakenly judged as belonging to that level; FN (False Negative) refers to the number of samples that actually belong to a certain level but are missed in judgment.

Accuracy reflects the overall classification correctness rate of the model. Precision measures the reliability of the model’s prediction results for a specific stress level; a higher value indicates fewer false positives. Recall, on the other hand, reflects the model’s ability to detect true stress samples; a higher value indicates fewer false negatives. Given that Precision and Recall may show a trade-off (where one increases at the expense of the other), the F1-Score, as the harmonic mean of the two, can effectively balance the model’s reliability and detection ability. It is particularly suitable for evaluating the model’s comprehensive discrimination performance across different salt stress levels.

## 3 Results and discussion

### 3.1 Experimental results and analysis of different degrees of salt stress

**Figure 10:**
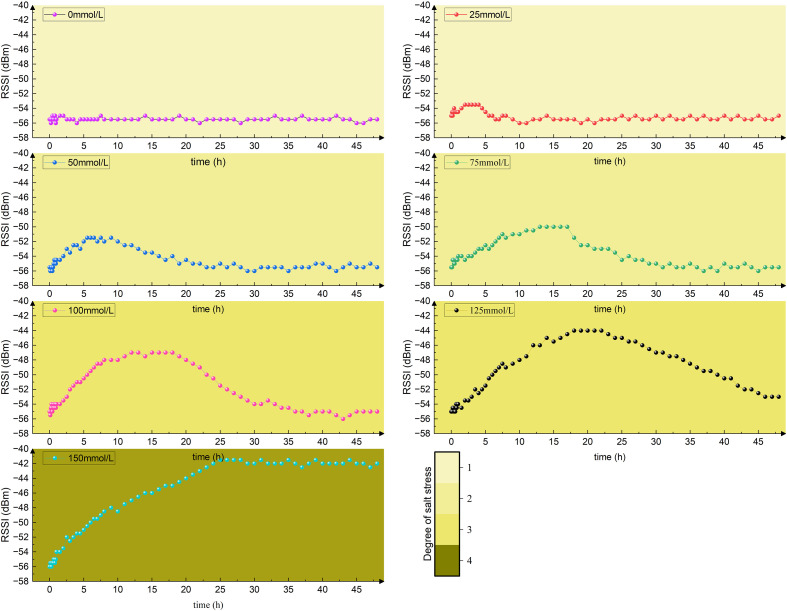
Changes in RSSI signal strength.

In the initial experiment applying a gradient concentration of NaCl solution to the roots of *Phyllostachys edulis*, the variation in RSSI signal strength is depicted in **Figure 10**. The baseline signal stabilized at approximately -55.4 dBm, with the untreated control group consistently maintaining relative stability.

When the salt concentration increased to 25 mmol/L, the signal strengthened to -53.5 dBm. This enhancement is primarily associated with an elevation in the leaf dielectric constant: the low-salt environment may increase ion concentration in the intercellular space/cytoplasm, optimizing the electrical conductivity of the tissue fluid and thereby enhancing the tissue’s conduction efficiency for electromagnetic waves.

As the salt concentration further increased to 50 mmol/L and 75 mmol/L, the signal continued to strengthen (reaching -51.5 dBm and -50 dBm, respectively). This reflects an increase in the real part of the complex permittivity of the leaves, where the transmembrane hydration layer structure maintained by cellular osmotic adjustment may further reinforce the dielectric properties.

When the salt concentration surpassed the 100 mmol/L threshold, the signal surged to a peak of -47 dBm, signifying a qualitative shift in the dielectric response: On one hand, high salt ion concentration significantly increased cytoplasmic electrical conductivity, leading to an elevation in the dielectric loss factor. On the other hand, physiological changes induced by stress, such as proline accumulation, may also influence the capacitive properties of cell membranes. These two factors act synergistically to significantly enhance the leaf’s energy coupling efficiency with electromagnetic waves.

Under the 125 mmol/L treatment, the signal further strengthened to -43.5 dBm. Dielectric spectroscopy characteristics at this stage revealed a leftward shift in relaxation frequency, reflecting reduced cell membrane fluidity or hindered molecular motion. Combined with the occurrence of membrane lipid peroxidation, this indicates that membrane structural damage has led to significant alterations in dielectric loss characteristics (such as peak broadening or anomalous intensity), approaching a limiting state under this stress level. Upon stress removal, the signal could only partially recover to around -52 dBm.

When the concentration reached 150 mmol/L, the signal rose to -41.5 dBm, indicating that the leaf dielectric properties entered a state of severe disruption. At this point, irreversible structural damage, including chloroplast disintegration and mitochondrial dysfunction, had occurred. Ultimately, the severe cellular structural damage constitutes the fundamental irreversible cause. This prevents the restoration of abnormal dielectric properties—resulting from the combined effects of structural damage and persistent ionic effects (such as sustained ionic shielding)—even after stress removal. Consequently, the RSSI signal cannot return to baseline levels.

**Figure 11:**
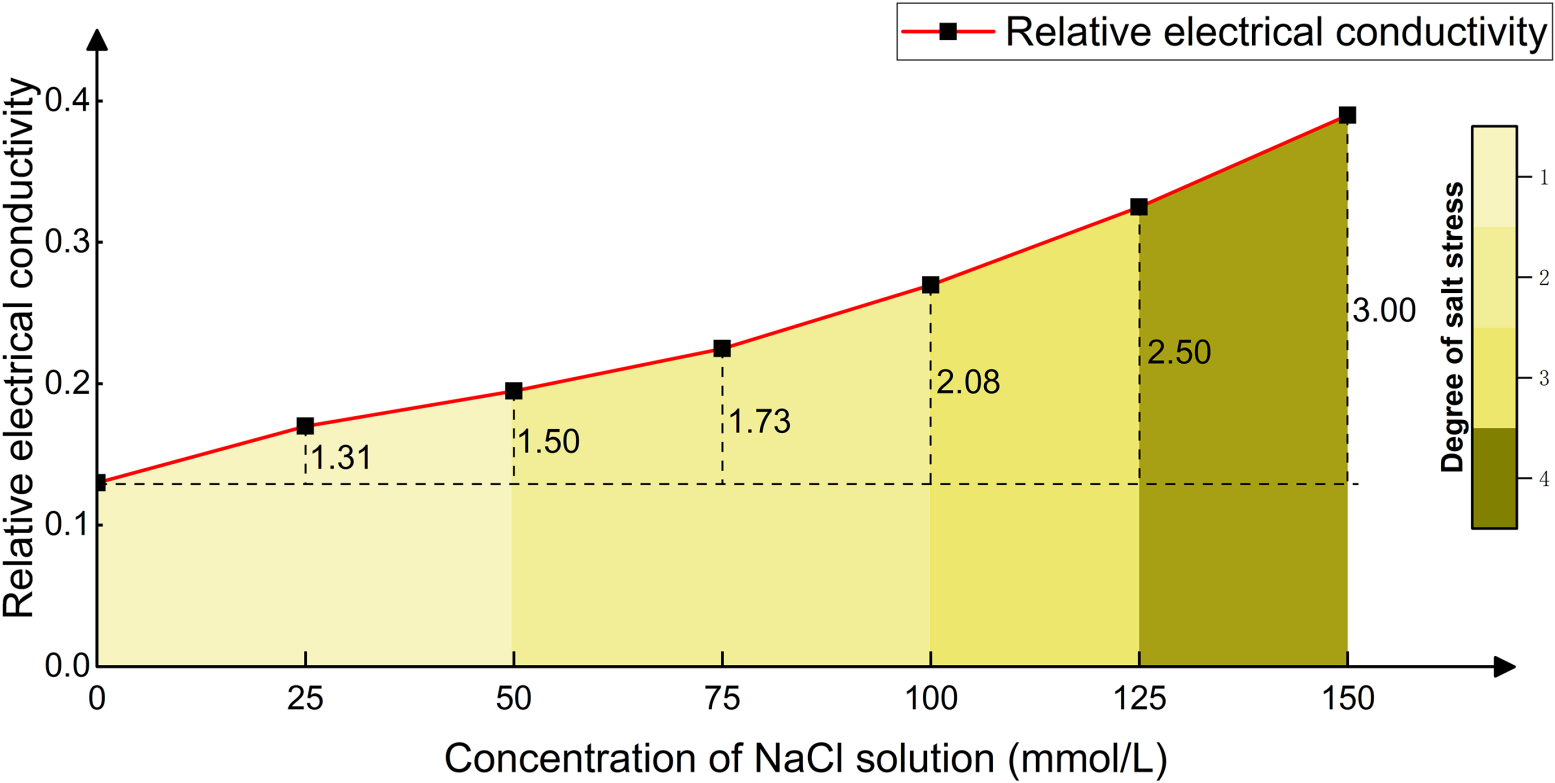
Relative conductivity of *Phyllostachys edulis* leaves.

**Figure 12:**
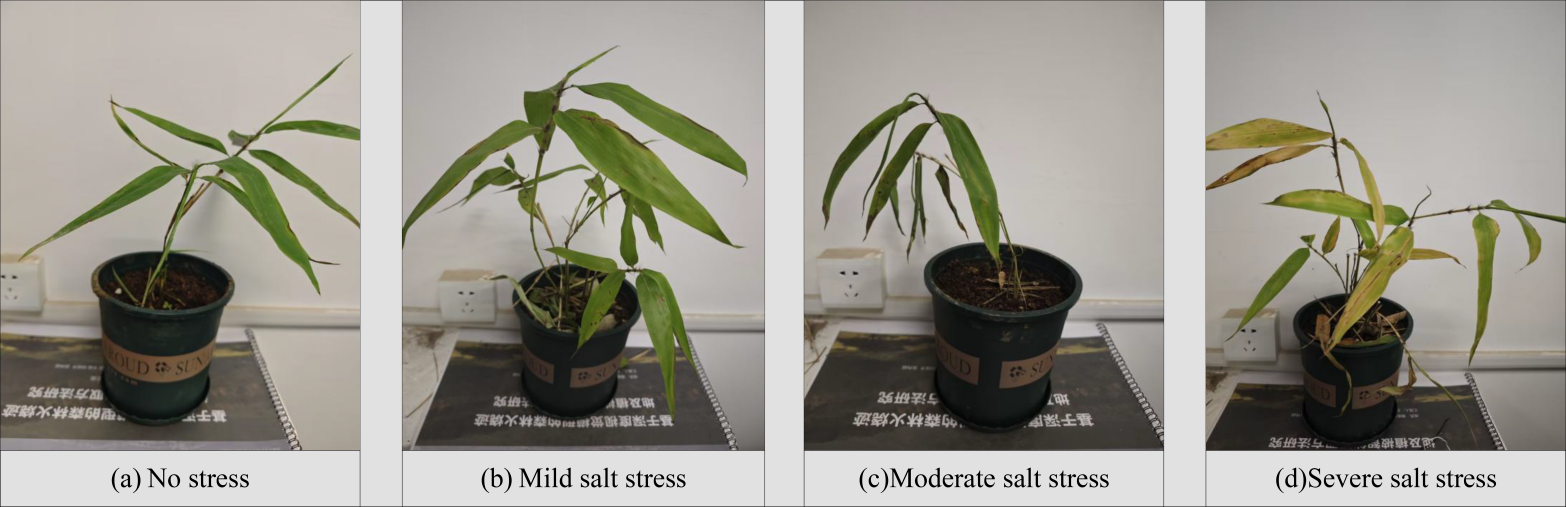
Phenotypic comparison of *Phyllostachys edulis* under different salt stress levels. **(a)** No stress: healthy, fully expanded green leaves. **(b)** Mild salt stress: slight leaf curling and initial wilting. **(c)** Moderate salt stress: pronounced wilting and chlorosis. **(d)** Severe salt stress: severe chlorosis, necrosis, and extensive leaf desiccation.

In seven groups of control experiments, *Phyllostachys edulis* was subjected to long-term treatment with NaCl solutions of different concentrations, and the changes in relative conductivity of its leaves are shown in **Figure 11**. As the concentration of NaCl solution increased from 0 mmol/L to 150 mmol/L, the relative conductivity of leaves showed a gradient increasing trend, reaching 1.31, 1.50, 1.73, 2.08, 2.50, and 3.00 times that of the control group, respectively. This indicates a significant positive correlation between salt concentration and the degree of cell membrane damage. In the concentration range of 0–50 mmol/L (including the 25 mmol/L and 50 mmol/L groups), the increase in relative conductivity did not exceed 50% (≤1.50 times). The cell membrane structure remained intact, and the electrolyte leakage did not reach the damage threshold. This range is defined as the non-salt stress state (color scale 1). When the concentration increased to 50–100 mmol/L (including the 75 mmol/L and 100 mmol/L groups), the relative conductivity increased to 1.73-2.08 times. At this point, osmotic adjustment substances (such as proline) began to accumulate, but the degree of membrane lipid peroxidation intensified, resulting in reversible cell damage, thus classified as mild salt stress (color scale 2). When the concentration reached 100–125 mmol/L (including the 125 mmol/L group), the relative conductivity jumped to 2.50 times. The selective barrier function of the cell membrane declined, and the ion homeostasis regulation mechanism collapsed, leading to massive leakage of intracellular solutes. This stage is categorized as moderate salt stress (color scale 3). When the concentration exceeded 125 mmol/L (150 mmol/L group), the relative conductivity soared to 3.00 times. The chloroplast structure disintegrated and reactive oxygen species accumulated explosively, marking the initiation of programmed cell death. At this point, *Phyllostachys edulis* was in an irreversible severe salt stress state (color scale 4). The growth status of *Phyllostachys edulis* under different stress levels after long-term treatment is shown in **Figure 12**, which are classified into non-stress, mild salt stress, moderate salt stress, and severe salt stress. This classification system provides a theoretical basis for the study of *Phyllostachys edulis*’s salt stress response mechanism.

### 3.2 Performance comparison of various optimization algorithms and standard machine learning classifiers

To comprehensively evaluate the comprehensive performance of the improved C-T-PSO algorithm, this section conducts experimental comparisons between it and four representative swarm intelligence optimization algorithms, including Particle Swarm Optimization (PSO) (Tang and Meng, 2024), Ant Colony Optimization (ACO) (Blum, 2024), Grey Wolf Optimizer (GWO) (Premkumar et al., 2024), Whale Optimization Algorithm (WOA) (Rajmohan et al., 2023), and Sparrow Search Algorithm (SSA) (Sun et al., 2023). By comparing these mainstream algorithms, the aim is to examine the relative advantages of C-T-PSO in solving optimization problems.

**Figure 13:**
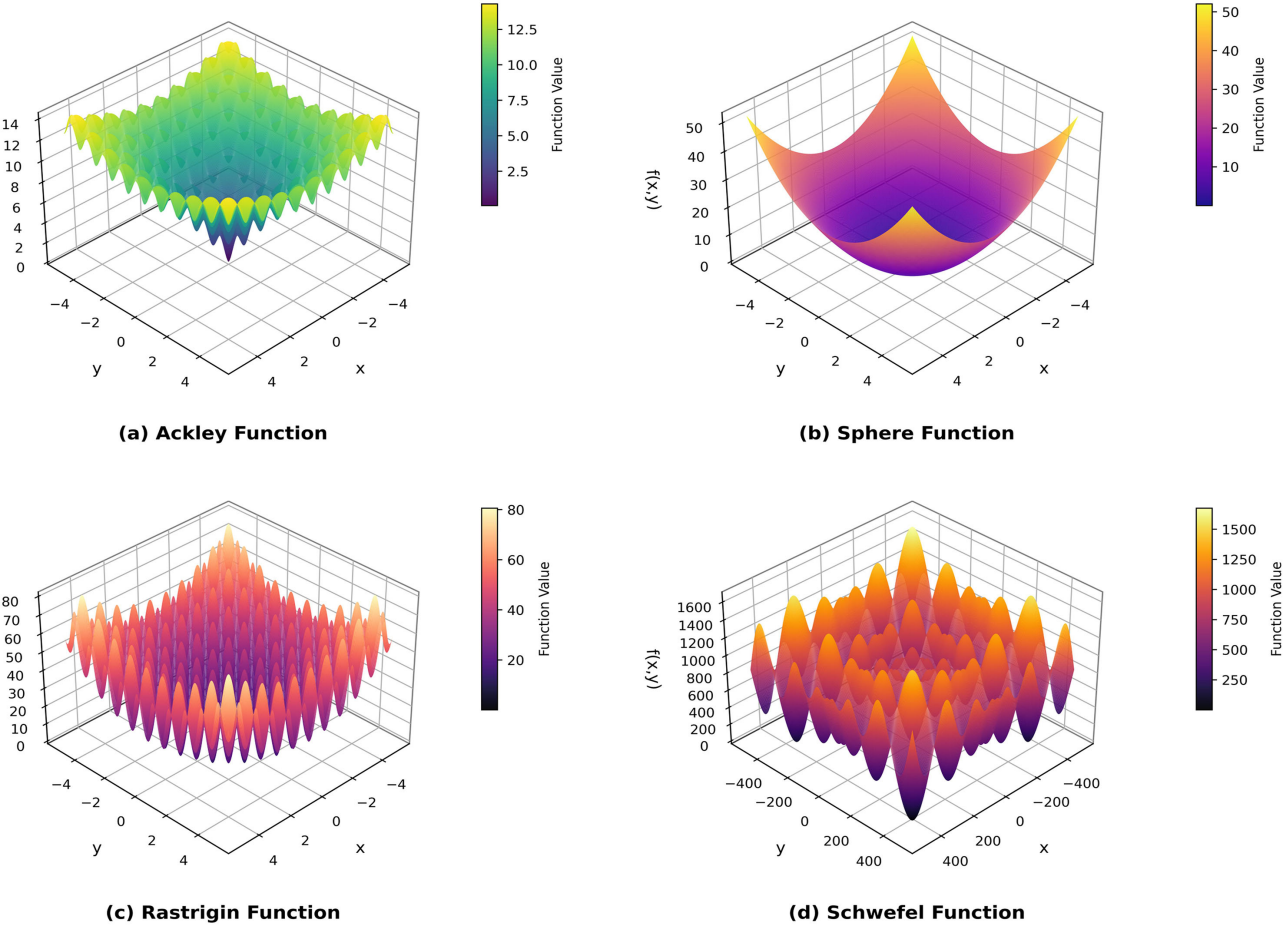
Four types of test functions. **(a)** Ackley Function. **(b)** Sphere Function. **(c)** Rastrigin Function. **(d)** Schwefel Function.

Meanwhile, to thoroughly investigate the robustness of the algorithm in coping with different optimization challenges, four types of benchmark test functions with typical structural characteristics are selected for evaluation. Among them, the Ackley and Sphere functions contain only one minimum value within their value ranges, belonging to unimodal optimization problems; while the Rastrigin and Schwefel functions have multiple minimum values, which tend to cause the algorithm to fall into local optima and are typical multimodal optimization problems. To explore the performance and optimization efficiency of the C-T-PSO algorithm after multi-strategy joint optimization in various functions, multiple test functions are selected for experiments, as shown in **Figure 13**. **Table 3** specifies the expressions, detailed parameters (including x value ranges), and minimum values of these four test functions.

**Table 3:** The detailed attributes of the selected test function.

Function name	Expression	x value range	Minimum value
Ackley	$\text{f(x) }=\text{ - }a\text{exp(-b}\sqrt{\frac{1}{\text{d}}}\text{)- exp(}\frac{1}{\text{d}}\sum_{i=1}^{\text{d}}{\text{cos(cx}}_{\text{i}})+a+e$	[-32.786,32.786]	0
Sphere	$\text{f}\left(\text{x}\right)=\sum_{\text{i}=1}^{\text{d}}{\text{x}}_{\text{i}}^{2}$	[-100,100]	0
Rastrigin	$\text{f}\left(\text{x}\right)=10\text{d}+\sum_{\text{i}=1}^{\text{d}}[{\text{x}}_{\text{i}}^{2}-10\cos\left(2{\text{πx}}_{\text{i}}\right)]$	[-5.12,5.12]	0
Schwefel	$\text{f}\left(\text{x}\right)=\;\sum_{\text{i}=1}^{\text{d}}{\left(\sum_{\text{j}=1}^{\text{i}}{\text{x}}_{\text{j}}\right)}^{2}$	[-500,500]	0

**Figure 14:**
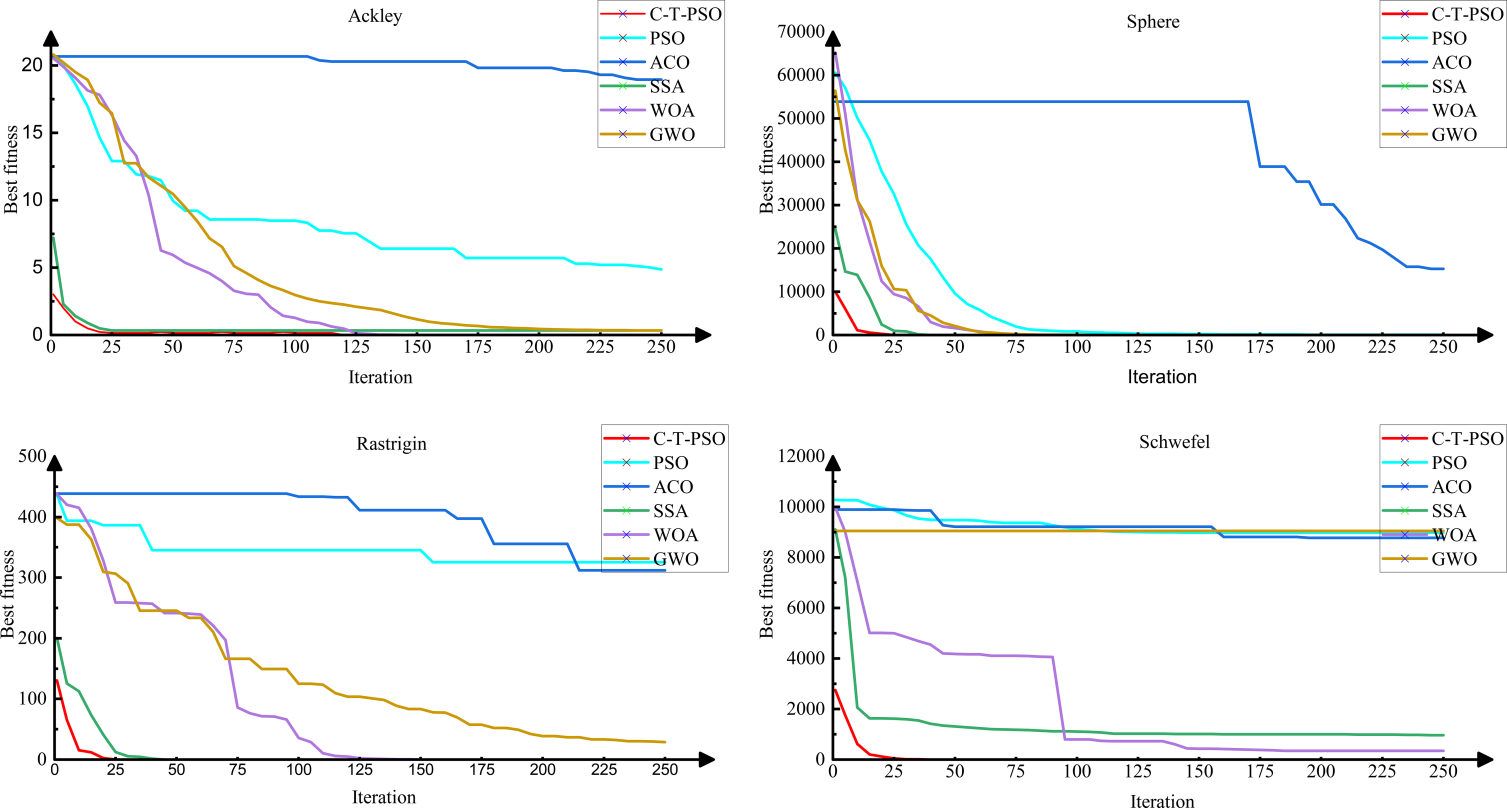
Convergence performance of six algorithms on selected test functions.

To control the impact of randomness, the initial population sizeZ and maximum number of iterations for all five algorithms were uniformly set to 50 and 250, respectively. The experimental results (**Figure 14**) show that the C-T-PSO algorithm proposed in this chapter outperforms other comparative algorithms in both convergence speed and convergence accuracy, with a faster convergence rate and the ability to find better solutions more quickly.

To quantitatively evaluate the convergence accuracy and robustness of the algorithms, each algorithm was independently executed 30 times on each test function. The statistical results (mean ± standard deviation) are presented in **Table 4**. The analysis demonstrates that the C-T-PSO algorithm achieved the best or competitive mean performance on the vast majority of functions. Moreover, its standard deviation is significantly lower than that of other comparative algorithms, proving its comprehensive advantages in high solution accuracy and strong stability. These statistical results corroborate the convergence trends observed in **Figure 14**.

**Table 4:** Statistical results (Mean ± Standard Deviation) of the optimization algorithms over 30 independent runs on benchmark functions.

Algorithm	Ackley	Sphere	Rastrigin	Schwefel
C-T-PSO	3.52×10^-7^ ± 1.21×10^-7^	3.39×10^-59^ ± 8.47×10^-60^	1.02×10^-7^ ± 0.2175	0.9812 ± 0.2451
PSO	0.0124 ± 0.0056	4.8754 ± 1.2243	325.3263 ± 81.4863	8970.6163 ± 2240.7352
ACO	0.8761 ± 0.2343	15300.6734 ± 3830.6489	312.5765 ± 78.1637	8780.2489 ± 2190.3243
SSA	0.3271 ± 0.0523	1.95×10^-36 ±^ 4.88×10^-47^	0.0032 ± 2.0574	963.7134 ± 241.1244
WOA	0.0016 ± 8.17×10^-4^	5.78×10^-6 ±^ 1.44×10^-6^	1.92×10^-5 ±^ 95.8784	341.4241 ± 85.2746
GWO	0.3383 ± 0.0612	0.8485 ± 0.2121	28.9867 ± 7.2383	9050.7873 ± 2260.6453

To further systematically evaluate the additional value and comparative advantages of the proposed physics-model-driven approach, a comprehensive comparison was conducted with three classical machine learning algorithms that directly utilize raw RFID signals for classification. The models selected for comparison were carefully chosen to cover different machine learning paradigms: Support Vector Machine (SVM) as a representative of powerful linear and nonlinear classifiers; Random Forest (RF) as an efficient and robust ensemble learning algorithm; and XGBoost as a top-performing gradient boosting framework renowned for its excellence in numerous data science competitions.

### 3.3 Determination and analysis of salt stress diagnosis results for *Phyllostachys edulis*

**Figure 15:**
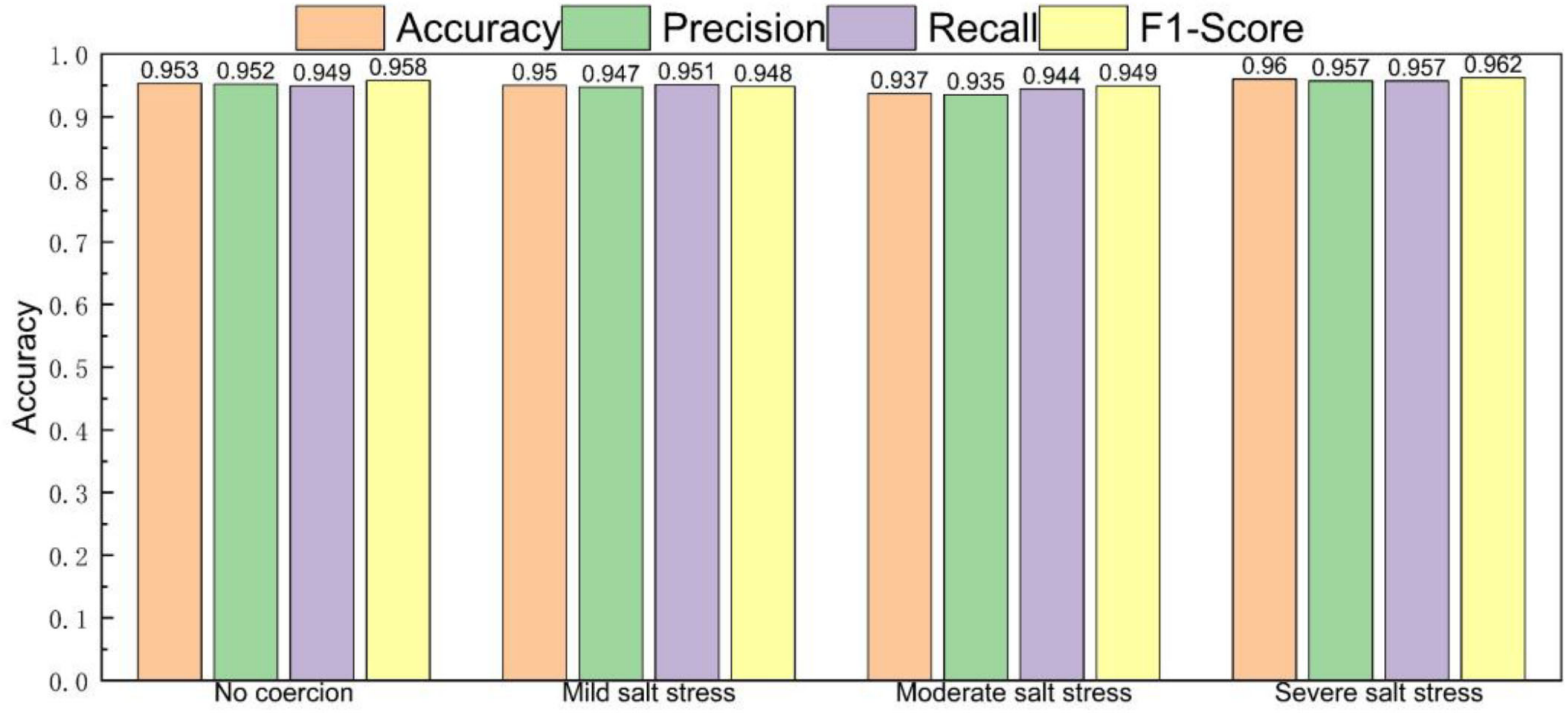
Multi-indicator performance evaluation of gradient salt stress.

**Figure 16:**
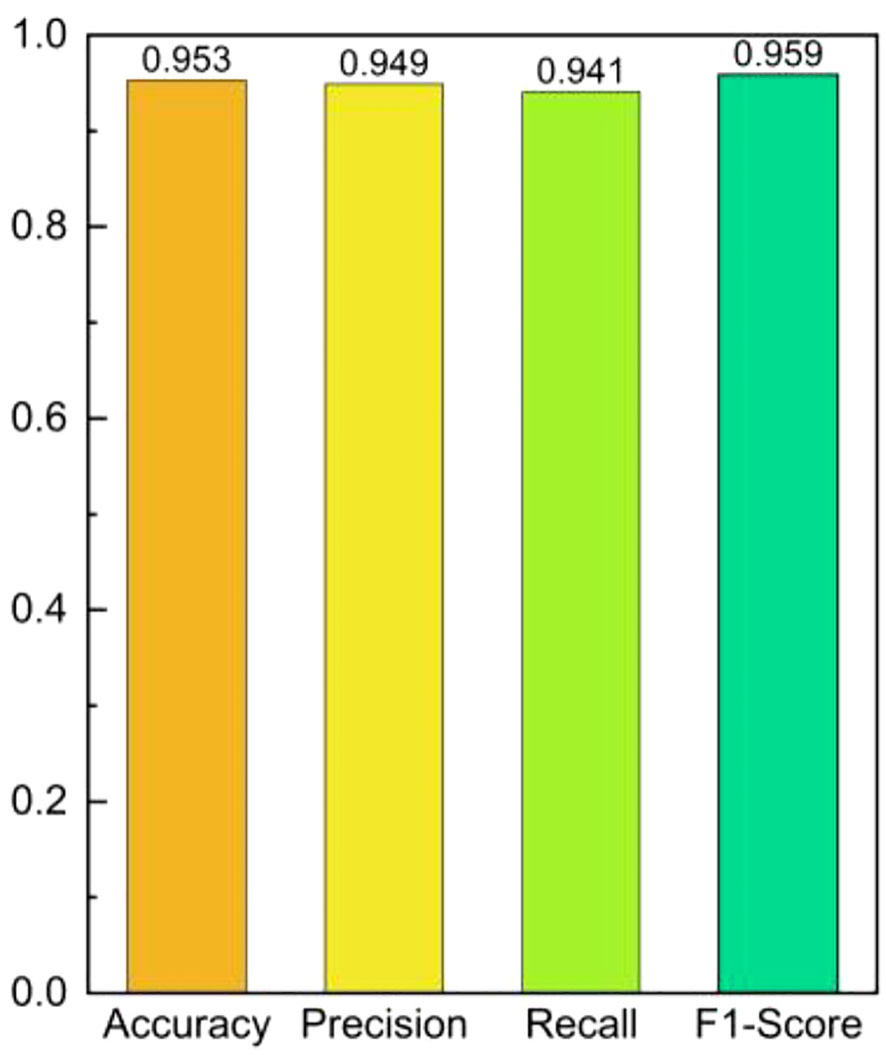
Overall performance evaluation of multi-indicator for salt stress.

The experimental results are shown in **Figure 15**. The C-T-PSO-Cole-Cole model constructed in this study exhibits comprehensively excellent performance in the detection of salt stress in *Phyllostachys edulis*. For the four states of no stress, mild salt stress, moderate salt stress, and severe salt stress, the core evaluation indicators of the model—Accuracy, Precision, Recall, and F1-Score—all stably exceed 93%. This data indicates that the model has a high generalization ability in classifying samples with different stress levels. The synchronous optimization of its Precision and Recall ensures the reliability of the prediction results, while the F1-Score verifies the overall advantage of the classification effectiveness. The comprehensive performance evaluation of the overall experimental results is shown in **Figure 16**. The model demonstrates excellent stability in the global dimension: the core indicators (Accuracy, Precision, Recall, and F1-Score) all remain consistently in the high range of over 94%.

**Figure 17:**
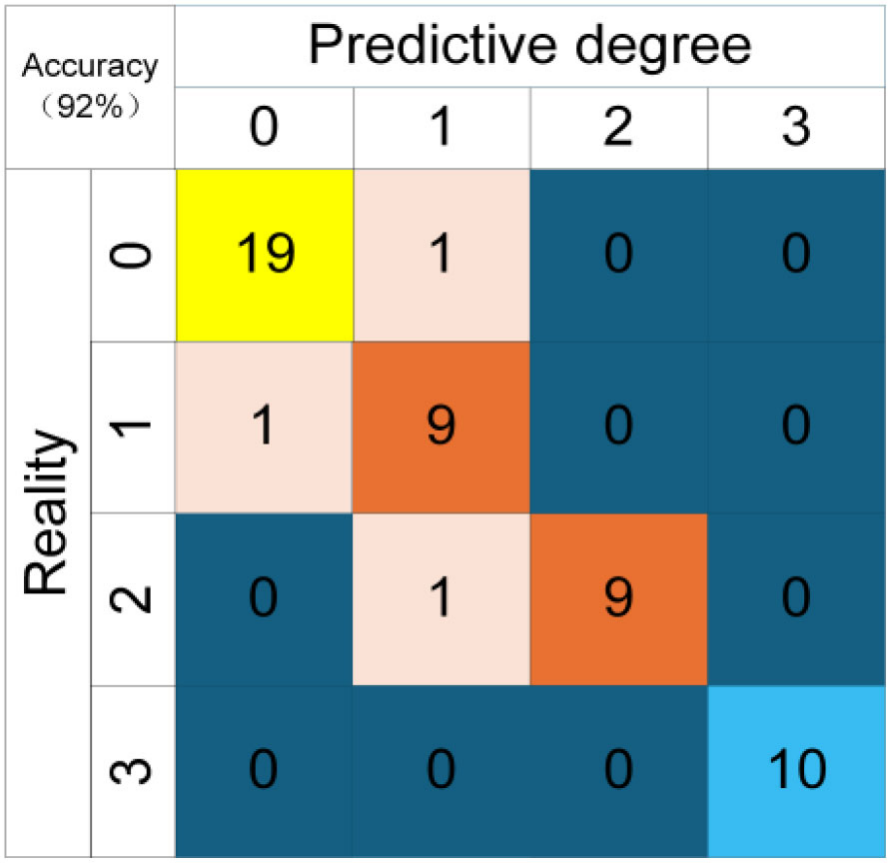
Confusion matrix based feature layer fusion.

**Figure 17** shows the confusion matrix based on feature layer fusion. After selecting 50 test samples for prediction, their relative conductivity was measured immediately, including 20 samples with no stress (0), 10 samples with mild salt stress (1), 10 samples with moderate salt stress (2), and 10 samples with severe salt stress (3). The prediction results indicate that 1 no-stress sample was misjudged as mild stress, 1 mild salt stress sample was misjudged as no stress, 1 moderate salt stress sample was misjudged as no stress, and all severe salt stress samples were predicted correctly. The prediction results are basically consistent with the experimental data of relative conductivity.

**Figure 18:**
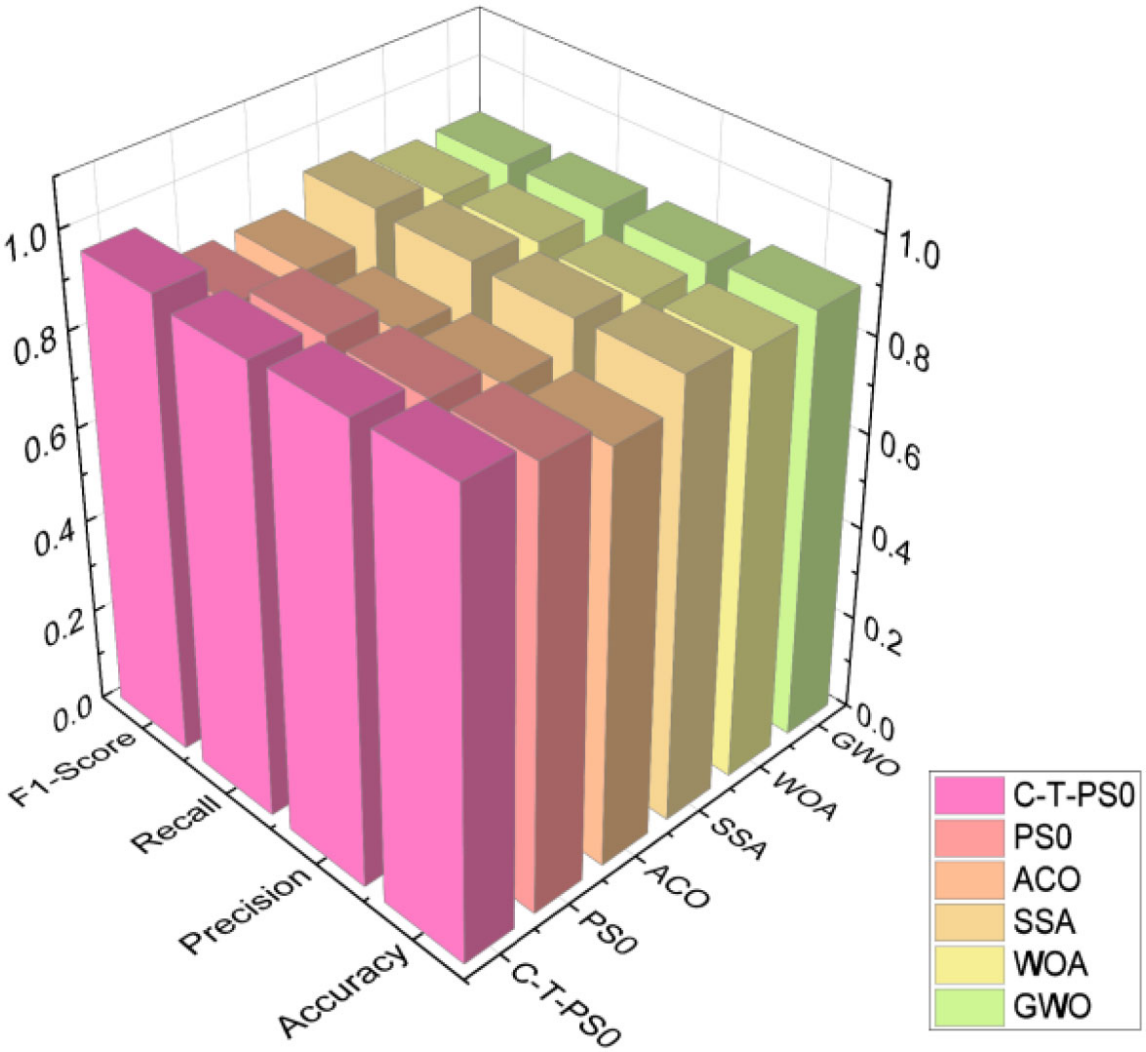
Performance comparison of six optimization algorithms.

**Figure 19:**
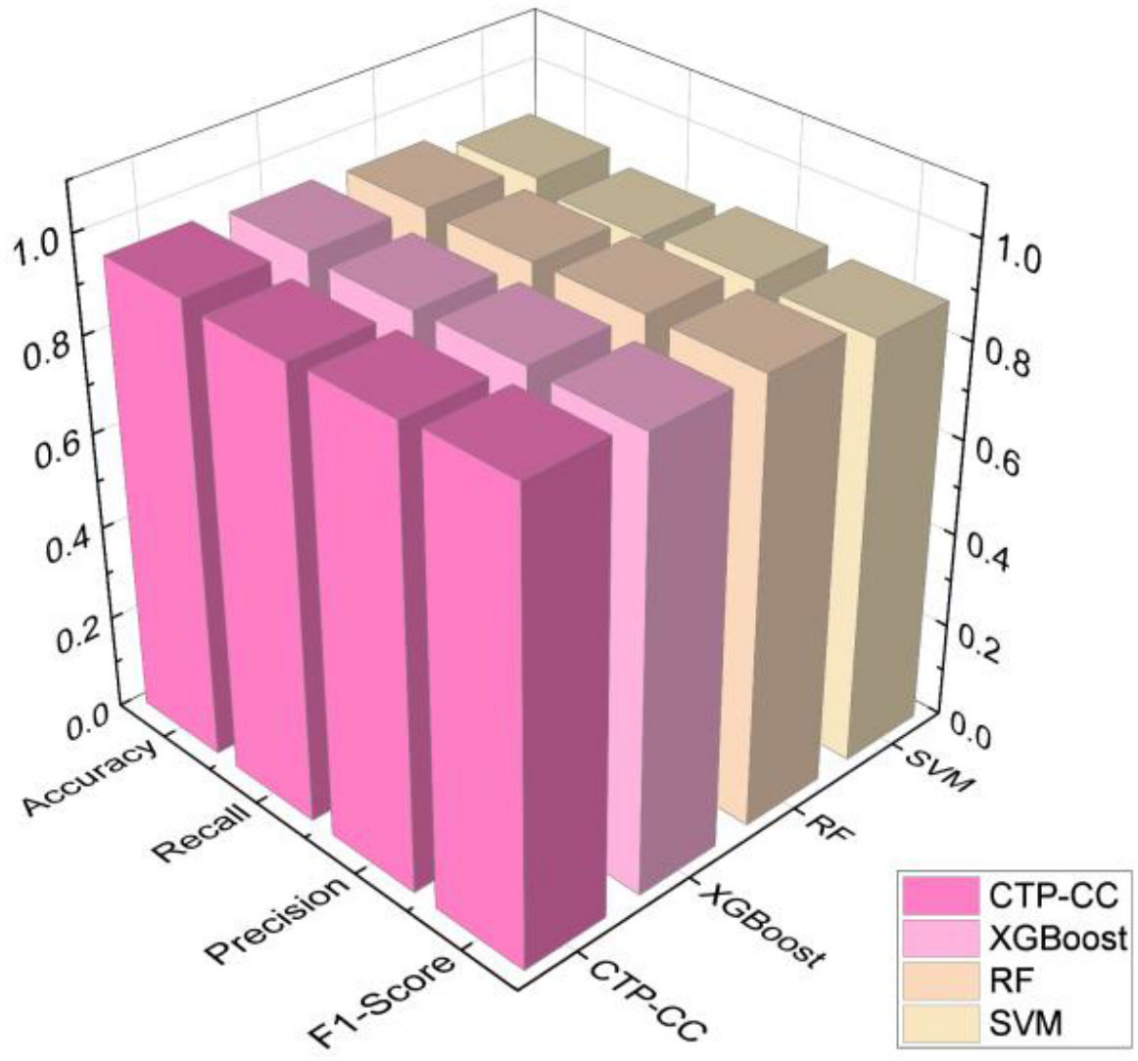
Performance comparison of standard machine learning classifiers.

**Figure 18** shows the experimental results of diagnosing the salt stress status of *Phyllostachys edulis* after inverting the Cole-Cole model parameters using five optimization models. From the comprehensive comparison of various evaluation indicators in the figure, it can be clearly observed that the C-T-PSO-Cole-Cole model constructed in this study exhibits significant advantages in all key performance indicators. Compared with the other five comparative methods, this model not only achieves the highest comprehensive score but also shows higher reliability and stability in identifying different stress levels. The performance comparison between the C-T-PSO-Cole-Cole model and standard machine learning classifiers is shown in **Figure 19**. All comparative models demonstrated commendable performance, with XGBoost achieving an accuracy of 94.2%, which confirms the effectiveness of raw RFID features for salt stress diagnosis. The proposed C-T-PSO-Cole-Cole model achieved optimal results across all metrics, exhibiting marginal yet consistent improvements over XGBoost with an accuracy of 95.3% and an F1-score of 95.9%. This result strongly confirms that the *Phyllostachys edulis* salt stress diagnosis method based on the optimized C-T-PSO-Cole-Cole model has excellent performance and practical value. It provides a more effective technical solution for precise non-destructive monitoring of *Phyllostachys edulis* salt stress and a robust analysis tool for plant stress response research.

## 4 Conclusion

This paper proposes a non-destructive detection method for salt stress in *Phyllostachys edulis* based on UHF RFID signal analysis and the C-T-PSO-Cole-Cole model. In a laboratory environment, by reading the reflected signals from passive RFID tags attached to the abaxial surface of *Phyllostachys edulis* leaves under different gradient salt stresses, radio frequency features such as RSSI and phase were collected, and their dynamic response patterns with varying stress intensities were quantified. Based on the Cole-Cole dielectric model, a nonlinear mapping relationship between the dielectric properties of *Phyllostachys edulis* leaves and the degree of salt stress was established, and the physiological interpretation of the model parameters was clarified. Furthermore, the Chebyshev chaotic mapping was introduced to initialize the particle swarm, enhancing the global search capability through high-dispersion initial solutions and avoiding local optimal traps. Finally, combined with the t-distribution perturbation strategy to dynamically adjust the inertia weight, the exploration and exploitation capabilities were adaptively balanced during the iteration process, significantly improving the accuracy of model parameters. To verify the generalization ability of the model, relevant data of several *Phyllostachys edulis* leaves were collected from the experimental forest base of Nanjing Forestry University. After data collection, the leaves were immediately sealed and sent to the laboratory, and the salt stress levels were accurately calibrated by the relative conductivity measurement method to verify the accuracy of the algorithm model. The experimental results show that the C-T-PSO-Cole-Cole model exhibits comprehensive advantages in salt stress diagnosis tasks: the classification accuracy (Accuracy = 0.953), precision (Precision = 0.949), recall (Recall = 0.941), and F1-score (F1-Score = 0.959) are all superior to those of the comparison models, verifying the effectiveness of the multi-stage optimization strategy. To further validate the robustness of the classification model, we re-evaluated it using 10-fold cross-validation. The results demonstrated that the model achieved an average classification accuracy of 94.5% (standard deviation: ± 1.2%). This outcome is highly consistent with the accuracy obtained from the initial train-test split (95.3%). More importantly, the exceptionally low standard deviation indicates stable performance across different data partitions, effectively mitigating concerns regarding potential randomness arising from a single random split. This strongly confirms that the C-T-PSO-Cole-Cole model exhibits remarkable and reliable generalization capability even with limited samples.

The above research verifies the core hypotheses proposed in the introduction: (1) Salt stress alters the osmotic pressure inside and outside the leaf cells of *Phyllostachys edulis*, which in turn affects their dielectric constant. This change can reflect the degree of salt stress by detecting the dielectric properties of leaves; (2) UHF radio frequency signals are highly responsive to changes in the dielectric constant of the medium. By quantifying the characteristics of radio frequency signals such as RSSI and phase, the dynamic changes in the dielectric properties of *Phyllostachys edulis* leaves can be inverted, thereby achieving accurate discrimination of salt stress levels.

Compared with the widely studied deep learning methods, the proposed approach exhibits unique advantages. Although CNN excels in processing image features and LSTM possesses inherent strengths in modeling time-series data, these methods—if applied to RFID signal analysis—may achieve good performance, but their “black-box” nature results in a lack of physical interpretability in the decision-making process.

The innovative value of our method lies in successfully establishing an interpretable linkage from RFID signals to plant physiological status. Through the inversion of Cole-Cole model parameters, we can directly correlate changes in dielectric properties with physiological processes such as ion balance and membrane integrity at the cellular level. This offers an analytical perspective with greater physical transparency for understanding plant stress response mechanisms compared to purely data-driven deep learning approaches. Furthermore, the computational efficiency of our method is significantly higher than that of complex deep learning models, making it more suitable for real-time processing on resource-constrained field monitoring devices.

It is noteworthy that this study does not aim to completely replace deep learning methods. Rather, the two technical pathways exhibit distinct advantages in different scenarios: deep learning performs exceptionally well in contexts with massive annotated data and no requirement for interpretability, while our method demonstrates greater practical value in situations with limited data, where interpretability is essential, and field deployment is needed.

While the proposed C-T-PSO-Cole-Cole model demonstrates high accuracy under controlled laboratory conditions, its performance in complex and unstructured field environments may be challenged by various sources of uncontrolled noise and environmental variability. Key confounding factors include diurnal and seasonal fluctuations in temperature and humidity, which directly affect the dielectric properties of plant tissues and thus can masquerade as or obscure salt stress signals. Additionally, electromagnetic interference from other wireless devices and signal attenuation or multipath effects caused by rain, wind, and dense foliage could significantly bias the acquired RFID signal features, namely the Received Signal Strength Indicator and phase, potentially leading to misinterpretations of plant physiological status. To ensure the scalability and robustness of our method for practical real-world applications, we have identified two primary limitations and propose corresponding strategic solutions for future research and development.

1. To combat signal instability induced by fluctuating microclimatic conditions, we propose the development of a hybrid signal processing framework. This framework will synergistically combine time-frequency analysis techniques. Specifically, we will employ Wavelet Packet Transform for its multi-resolution analysis capabilities to isolate non-stationary noise components from the raw RFID signal. This will be coupled with an Adaptive Kalman Filter, which will dynamically adjust its parameters based on real-time inputs from integrated temperature and humidity sensors. This dual approach aims to differentiate between dielectric changes caused by environmental fluctuations and those arising from genuine salt stress, thereby significantly enhancing the signal-to-noise ratio and diagnostic fidelity in noisy field conditions.
2. Acknowledging the limitation of single-leaf detection in capturing whole-plant physiological status, our future work will focus on architecting a multi-modal, space-ground collaborative sensing network. At the canopy level, unmanned aerial vehicles equipped with multi-reader RFID arrays will perform systematic raster scans to map spatial variance in stress responses. At the plant level, a network of ultra-thin, flexible RFID sensor tags will be deployed on multiple leaves and branches to continuously monitor stress propagation dynamics. The data fusion engine will integrate this multi-source RFID data with hyperspectral imagery acquired from the UAVs. This integration will leverage machine learning models, particularly convolutional neural networks for feature extraction from images and recurrent neural networks for time-series analysis of RFID data, to construct a spatiotemporally explicit model for visualizing, forecasting, and understanding the systemic dynamics of salt stress in *Phyllostachys edulis*.

This study provides technical support for measuring the salt stress level of *Phyllostachys edulis*. In the future, through hardware innovation and algorithm iteration, the monitoring efficiency and application scope will be further enhanced.

## Data Availability

The original contributions presented in the study are included in the article/supplementary material. Further inquiries can be directed to the corresponding author.

## References

[B1] Awal M. A. Naqvi S. A. R. Abbosh A. (2024). “Integrating dielectric modelling with machine learning for skin cancer classification,” in Proc. IEEE Asia-Pacific Microwave Conf. (APMC) 2024. 907–909 (New York, NY, USA: IEEE). doi: 10.1109/APMC60911.2024.10867598

[B2] Azimi S. Wadhawan R. Gandhi T. K. (2021). Intelligent monitoring of stress induced by water deficiency in plants using deep learning. IEEE Trans. Instrum. Meas. 70, 1–10. doi: 10.1109/TIM.2021.3111994 33776080

[B3] Benbaghdad M. Fergani B. Tedjini S. (2016). Backscatter signal model of passive UHF RFID tag: Application to collision detection. Electron. Letters. 52, 974–975. doi: 10.1049/el.2015.3974

[B4] Blum C. (2024). Ant colony optimization: A bibliometric review. Phys. Life Rev. 51, 87–95. doi: 10.1016/j.plrev.2024.09.014, PMID: 39341089

[B5] Boshimeniuci Jiang Y. Y. Li X. H. Song C. C. Fu Q. C. (2022). Study on effects of salt stress on physiological characteristics of Bambusa multiplex. Modern Horticulture. 45, 1–3. doi: 10.14051/j.cnki.xdyy

[B6] Cao Y. F. Yan R. Y. Sun M. C. Guo J. Zhang S. Y. (2025). Effects of exogenous chitosan concentrations on photosynthesis and functional physiological traits of hibiscus under salt stress. BMC Plant Biol. 25. doi: 10.1186/s12870-025-06424-x, PMID: 40181276 PMC11967025

[B7] Chetry M. Feng R. L. Babar S. Sun H. Zafar I. Mohany M.. (2025). Early detection and analysis of accurate breast cancer for improved diagnosis using deep supervised learning for enhanced patient outcomes. PeerJ Comput. Sci. 11. doi: 10.7717/peerj-cs.2784, PMID: 40567684 PMC12190644

[B8] Costa F. Genovesi S. Borgese M. Michel A. Dicandia F. A. Manara G. A. (2021). Review of RFID sensors, the new frontier of internet of things. Sensors 21. doi: 10.3390/s21093138, PMID: 33946500 PMC8124958

[B9] Daskalakis S. N. Assimonis S. D. Goussetis G. Tentzeris M. M. Georgiadis A. (2019). “The future of backscatter in precision agriculture,” in USNC-URSI Radio Science Meeting/IEEE International Symposium on Antennas and Propagation (AP-S). 647–648 (New York, NY, USA: IEEE). doi: 10.1109/apusncursinrsm.2019.8889330

[B10] Du J. Chen X. J. Wang Z. Nie X. H. Chen Y. C. Ai W. S.. (2024). Analysis of salt tolerance mechanism of Bambusa distegia based on transcriptome and metabolome. J. Hunan Agric. Univ. (Natural Sci. Edition). 50, 35–41. doi: 10.13331/j.cnki.jhau

[B11] Fiandaca G. Madsen L. M. Maurya P. K. (2018). Re-parameterisations of the Cole-Cole model for improved spectral inversion of induced polarization data. Near Surface Geophysics. 16, 385–399. doi: 10.3997/1873-0604.2017065

[B12] Fu C. Fu Q. Wang S. Wu F. Jiang N. Zhou R.. (2024). Genome-wide analysis of fatty acid desaturase genes in *Phyllostachys edulis* (*Phyllostachys edulis*) reveal their important roles in abiotic stresses responses. BMC Genomics 25. doi: 10.1186/s12864-024-11065-9, PMID: 39587486 PMC11590352

[B13] Hillier A. J. R. Makarovaite V. Gourlay C. W. Holder S. J. Batchelor J. C. A. (2019). Passive UHF RFID dielectric sensor for aqueous electrolytes. IEEE Sensors J. 19, 5389–5395. doi: 10.1109/JSEN.2019.2909353

[B14] Kamarudin M. H. Ismail Z. H. Saidi N. B. Hanada K. (2023). An augmented attention-based lightweight CNN model for plant water stress detection. Appl. Intell. 53, 20828–20843. doi: 10.1007/s10489-023-04583-8

[B15] Lee S. H. Jin I. S. (2011). Interoperation of an UHF RFID reader and a TCP/IP device *via* wired and wireless links. Sensors. 11, 10664–10674. doi: 10.3390/s111110664, PMID: 22346665 PMC3274307

[B16] Li J. Zhu Q. Zhu J. Cheng Y. Jia Z. Lu F.. (2024). Inimitable 3D pyrolytic branched hollow architecture with multi-scale conductive network for microwave absorption. J. Materials Sci. Technology. 173, 170–180. doi: 10.1016/j.jmst.2023.06.066

[B17] Lin W. C. Lo C. J. Cheng M. L. Zeng R. J. Hsieh Y. Y. Hou P. R.. (2024). An off-chip capacitance biosensor based on improved cole-cole model for the detection of trimethylamine N-oxide in early cardiovascular disease. IEEE Sensors J. 24, 11515–11526. doi: 10.1109/JSEN.2024.3368409

[B18] Liu Y. Wu C. Hu X. Gao H. Wang Y. Luo H.. (2020). Transcriptome profiling reveals the crucial biological pathways involved in cold response in *Phyllostachys edulis* (*Phyllostachys edulis*). Tree Physiol. 40, 538–556. doi: 10.1093/treephys/tpz133, PMID: 31860727

[B19] Moghimi A. Yang C. Miller M. E. Kianian S. F. Marchetto P. M. A. (2018). Novel approach to assess salt stress tolerance in wheat using hyperspectral imaging. Front. Plant Sci. 9. doi: 10.3389/fpls.2018.01182, PMID: 30197650 PMC6117507

[B20] Premkumar M. Sinha G. Ramasamy M. D. Sahu S. Subramanyam C. B. Sowmya R.. (2024). Augmented weighted K-means grey wolf optimizer: An enhanced metaheuristic algorithm for data clustering problems. Sci. Rep. 14. doi: 10.1038/s41598-024-55619-z, PMID: 38443569 PMC10914809

[B21] Rajmohan S. Elakkiya E. Sreeja S. R. (2023). Multi-cohort whale optimization with search space tightening for engineering optimization problems. Neural Computing Applications. 35, 8967–8986. doi: 10.1007/s00521-022-08139-8

[B22] Rubio A. Barbaro A. Montalvo G. Ortega-Ojeda F. E. García-Ruiz C. (2025). Influence of external light on ultra-weak photon emission of fruits: forensic differentiation of organic and conventional fruits. Sensors 25. doi: 10.3390/s25061799, PMID: 40292957 PMC11946304

[B23] Sharif A. Ouyang J. Raza A. Imran M. A. Abbasi Q. H. (2019). Inkjet-printed UHF RFID tag based system for salinity and sugar detection. Microwave Optical Technol. Letters. 61, 2161–2168. doi: 10.1002/mop.31863

[B24] Singhal R. K. Saha D. Skalicky M. Mishra U. N. Chauhan J. Behera L. P.. (2021). Crucial cell signaling compounds crosstalk and integrative multi-omics techniques for salinity stress tolerance in plant. Front. Plant Sci. 12. doi: 10.3389/fpls.2021.670369, PMID: 34484254 PMC8414894

[B25] Stehlik M. Chen P.-Y. Wong W. K. Kiselak J. A. (2024). Double Exponential Particle Swarm Optimization with non-uniform variates as stochastic tuning and guaranteed convergence to a global optimum with sample applications to finding optimal exact designs in biostatistics. Appl. Soft Comput. 163. doi: 10.1016/j.asoc.2024.111913

[B26] Steinhorst L. He G. Moore L. K. Schültke S. Schmitz-Thom I. Cao Y.. (2022). Ca2+-sensor switch for tolerance to elevated salt stress in Arabidopsis. Dev. Cell 57. doi: 10.1016/j.devcel.2022.08.001, PMID: 36007523

[B27] Sun Y. C. Luo L. Lin H. Lin D. M. Lin Z. X. (2022). Transcriptome analysis of Arundo donax stems in response to salt stress. Southwest China J. Agric. Sci. 35, 2708–2718. doi: 10.16213/j.cnki.scjas

[B28] Sun L. Si S. Ding W. Wang X. Xu J. (2023). Multiobjective sparrow search feature selection with sparrow ranking and preference information and its applications for high-dimensional data. Appl. Soft Computing 147. doi: 10.1016/j.asoc.2023.110837

[B29] Tang K. Z. Meng C. J. (2024). Particle swarm optimization algorithm using velocity pausing and adaptive strategy. Symmetry-Basel 16. doi: 10.3390/sym16060661

[B30] Wang J. J. Sun Q. Shang J. L. Zhang J. H. Wu F. Zhou G. S.. (2020). New approach for estimating soil salinity using A low-cost soil sensor *in situ*: A case study in saline regions of China's east coast. Remote Sens. 12. doi: 10.3390/rs12020239

[B31] Wang L. Zhang H. C. Bian L. M. Zhou L. Wang S. Y. Ge Y. F. (2024). Poplar seedling varieties and drought stress classification based on multi-source, time-series data and deep learning. Ind. Crop Prod. 218, 118905. doi: 10.1016/j.indcrop.2024.118905

[B32] Wu Y. Hou Z. Liu Y. Liu W. (2024). Leaf moisture content detection method based on UHF RFID and hyperdimensional computing. Forests 15. doi: 10.3990/f15101798

[B33] Wu L. G. Jiang Q. F. Zhang Y. Du M. H. Ma L. Ma Y. (2022). Peroxidase activity in tomato leaf cells under salt stress based on micro-hyperspectral imaging technique. Horticulturae 8. doi: 10.3390/horticulturae8090813

[B34] Xie L. B. Zhou M. Wang Y. Nie W. Yang X. L. Liu X. (2024). A low power clock generator with self-calibration for UHF RFID tags in intelligent terrestrial sensor networks. Wireless Networks. 30, 3409–3417. doi: 10.1007/s11276-019-02102-7

[B35] Zhang Y. Chai X. Cao L. Gan Z. Lu Y. Xie X. (2025). Exploiting 2D improved Sine-Chebyshev chaotic map and adaptive cellular automata permutation for image encryption. J. Mod Opt. 72, 741–766. doi: 10.1080/09500340.2025.2519795

[B36] Zhang J. C. Lu M. Zhou H. Du X. H. Du X. (2022). Assessment of salt stress to arabidopsis based on the detection of hydrogen peroxide released by leaves using an electrochemical sensor. Int. J. Mol. Sci. 23. doi: 10.3390/ijms232012502, PMID: 36293359 PMC9604455

[B37] Zhang J. Wei Z. Sun J. Guo M. Huang X. Chen X.. (2021). “Plant keeper: towards wireless sensing to ion transmission of plant,” in Proceedings of the 1st ACM Workshop on No Power and Low Power Internet-of-Things. 14–20 (New York, NY, USA: ACM). doi: 10.1145/3477085.3478990

[B38] Zhao S. Zhang Q. Liu M. Zhou H. Ma C. Wang P. (2021). Regulation of plant responses to salt stress. Int. J. Mol. Sci. 22, 4609. doi: 10.3390/ijms22094609, PMID: 33924753 PMC8125386

[B39] Zhou M. G. Ju F. E. Sun Z. H. Xu J. H. (2019). Analysis and research on characteristics of plant electrical signals under drought stress. J. Xi'an Univ. Technology. 35, 320–326+332. doi: 10.19332/j.cnki.jssn

[B40] Zhu Z. Li C. Du J. Wang B. Cao Y. Hu S. (2025). Genome-wide identification of the PeNHX gene family in *Phyllostachys edulis* and their responses to salt stress. Jiangsu Agric. Sci. 53, 169–177. doi: 10.15889/j.issn.1002-1302.2025.04.020

